# Fasting regulates mitochondrial function through lncRNA PRKCQ-AS1-mediated IGF2BPs in papillary thyroid carcinoma

**DOI:** 10.1038/s41419-023-06348-0

**Published:** 2023-12-14

**Authors:** Xiaoping Zhang, Yong Zhong, Lin Liu, Chengyou Jia, Haidong Cai, Jianshe Yang, Bo Wu, Zhongwei Lv

**Affiliations:** 1 Guangdong Provincial Key Laboratory of Tumor Interventional Diagnosis and Treatment, Zhuhai People’s Hospital, Zhuhai hospital Affiliated with Jinan University, Jinan University, 519000 Guangdong, China; 2 Department of Nuclear Medicine, Shanghai Tenth People’s Hospital, Tongji University, 200072 Shanghai, China; 3 Center of Thyroid, Department of General Surgery, Shanghai Jiao Tong University Affiliated Sixth People’s Hospital, 200233 Shanghai, China

**Keywords:** Cancer metabolism, Tumour-suppressor proteins

## Abstract

Recurring evidence suggests that fasting has extensive antitumor effects in various cancers, including papillary thyroid carcinoma (PTC). However, the underlying mechanism of this relationship with PTC is unknown. In this study, we study the effect of fasting on glycolysis and mitochondrial function in PTC. We find that fasting impairs glycolysis and reduces mitochondrial dysfunction in vitro and in vivo and also fasting in vitro and fasting mimicking diets (FMD) in vivo significantly increase the expression of lncRNA-protein kinase C theta antisense RNA 1 (PRKCQ-AS1), during the inhibition of TPC cell glycolysis and mitochondrial function. Moreover, lncRNA PRKCQ-AS1 was significantly lower in PTC tissues and cells. In addition, PRKCQ-AS1 overexpression increased PTC cell glycolysis and mitochondrial function; PRKCQ-AS1 knockdown has the opposite effect. On further mechanistic analysis, we identified that PRKCQ-AS1 physically interacts with IGF2BPs and enhances protein arginine methyltransferases 7 (PRMT7) mRNA, which is the key player in regulating glycolysis and mitochondrial function in PTC. Hence, PRKCQ-AS1 inhibits tumor growth while regulating glycolysis and mitochondrial functions via IGF2BPs/PRMT7 signaling. These results indicate that lncRNA PRKCQ-AS1 is a key downstream target of fasting and is involved in PTC metabolic reprogramming. Further, the PRKCQ-AS1/IGF2BPs/PRMT7 axis is an ideal therapeutic target for PTC diagnosis and treatment.

## Introduction

Papillary thyroid carcinoma (PTC) is the most prevalent type of thyroid carcinoma, which is a common type of cancer with a high incidence rate among women [1, 2]. Prognosis for PTC has remained at ten years with the application of traditional surgical treatment options [3]. Consistent recurrence and metastasis necessitate the identification of new potential biological markers for PTC.

Calorie restriction (CR) has recently attracted a lot of attention due to its role in cancer treatment [4, 5]. Fasting mimicking diets (FMD) is a less aggressive type of CR [6–8], which have been considered as a treatment strategy in conjugation with chemotherapy [9]. During tumorigenesis, cells become more dependent on aerobic glycolysis rather than mitochondrial oxidative phosphorylation, allowing them to meet the energy and nutrient demands of actively proliferating cancer cells. Studies have indicated that FMD changes this dependency on glycolysis and decreases the proliferation of cancer cells, thus making FMD a potential treatment strategy for cancer [10, 11]. However, no study has been published to date on the underlying molecular effects of FMD on PTC.

Long non-coding RNA (lncRNA), a group of non-coding RNAs of approximately 200 bp length, interact with other RNAs or proteins and play important roles in transcriptional, post-transcriptional regulation and chromatin remodeling [12–14]. Previously, studies have identified dysregulation of lncRNA-protein kinase C theta antisense RNA 1 (PRKCQ-AS1) to be associated with cancer progression, including colorectal cancer, triple-negative breast cancer [15–17]. In the initial phase of the investigation, we accessed the Gene Expression Omnibus (GEO) database (GSE3678 and GSE3467) and observed that the expression of PRKCQ-AS1 in PTC is significantly diminished in the tissue adjacent to carcinoma. A recent study found that the PRKCQ-AS1 is highly upregulated in colorectal cancer patients [15], leading to an increase in proliferation and migration of CRC cells. However, there are no studies till date of lncRNA-PRKCQ-AS1’s role in PTC.

Insulin-like growth factor 2 binding proteins (IGF2BP 1, 2, and 3) are a family of evolutionarily conserved RNA-binding proteins known to be involved in many cellular processes such as proliferation, differentiation, migration, polarization, and metabolism [18]. Structurally, IGF2BP1, 2, and 3 appear similar to each other in the placement of the domains, with a molecular weight between 58 to 66 kDa [19]. With around 56% amino acid sequence similarity among the protein domains, they share at *N*-terminal two RNA-recognition motifs (RRMs) and four hnRNPK homology (KH) domains in the *C*-terminal. Studies based on in vitro flag tagged proteins have identified specifically IGF2BP1 binds to over 1000 mRNAs [20]. In many cancer types, IGF2BPs is identified to be highly expressed [21–24], however relatively less is known about its molecular and regulatory role in PTC.

Protein arginine methyltransferases (PRMTs) family of proteins catalyze the methyl transfer to arginine residues of protein substrates and have been linked to many types of cancer [25]. PRMT7 is one of PRMTs that could only catalyze formation of monomethyl arginine (Rme1) [26]. PRMT7 has been identified to play vital roles in breast and lung cancer by regulating metastasis and EMT transition [27, 28]. In this study, we identified that the lncRNA PRKCQ-AS1/IGF2BPs/PRMT7axis play an important role in PTC and specifically aimed at understanding their regulatory roles during fasting using in vitro and in vivo experimental models.

## Results

### Fasting impairs glycolysis and reduces mitochondrial dysfunction in vitro and in vivo

**Fig. 1:**
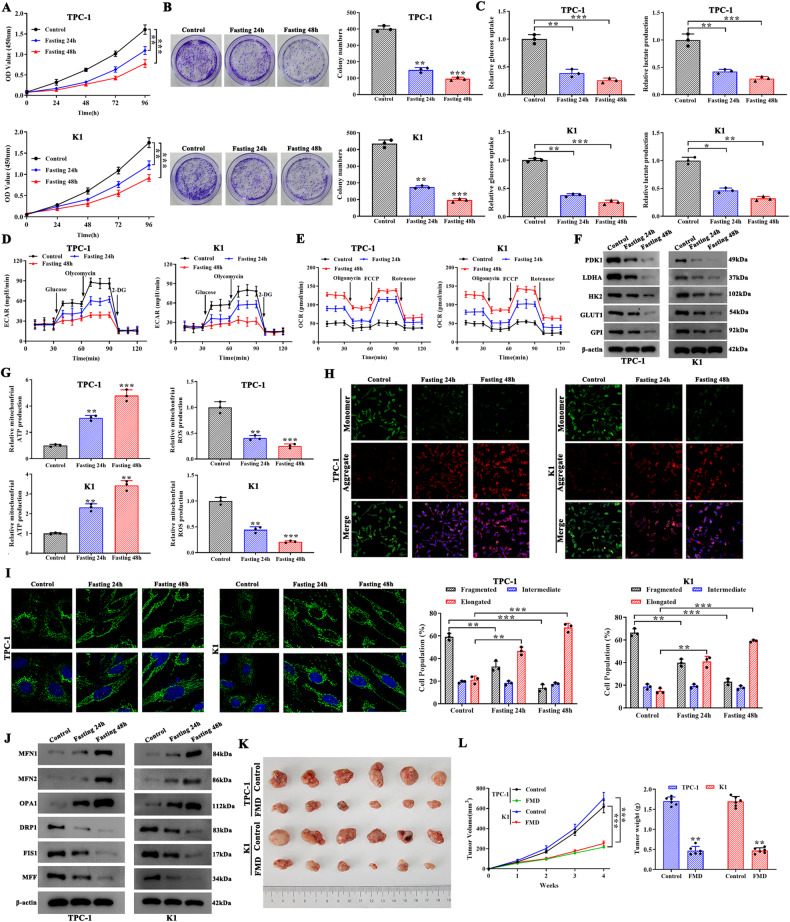
Fasting impairs glycolysis and reduces mitochondrial dysfunction of PTC cell lines in vitro and in vivo. **A**, **B** Fasting inhibited TPC-1 and K1 cell proliferation as measured by CCK8 and colony formation assay. **C** Fasting reduced glucose uptake and lactate production via glycolysis in TPC-1 and K1 cells. **D**, **E** ECAR, an indicator of glycolysis, was reduced, while OCR, which reflects mitochondrial respiration, was increased in TPC-1 and K1 cells cultured in the fasting mimic medium. **F** Fasting reduced the expression of rate-limiting glycolytic enzymes in glucose metabolism (PDK1, LDHA, HK2, GLUT1 and GPI) in TPC-1 and K1 cells, which were measured by western blotting. **G** Mitochondrial ROS and ATP production were measured in TPC-1 and K1 cells cultured in the fasting mimic medium. **H** JC-1 staining was used to observe mitochondrial membrane potential in TPC-1 and K1 cells cultured in the fasting mimic medium. Scale bars, 50 µm. **I** The proportion of cells with elongated, intermediate, and fragmented mitochondrial was quantified in TPC-1 cells cultured in the fasting mimic medium. Scale bars, 10 µm. **J** Western blot analysis for protein expression levels of MFN1, MFN2, OPA1, DRP1, FIS1, and MFF in TPC-1 and K1 cells cultured in the fasting mimic medium. **K** TPC-1 and K1 cells were injected into BALB/c mice. When the tumors were palpable, the mice were randomly assigned to the control group or the fasting mimic diet (FMD) group. Photograph of dissected tumors (*n* = 6) were shown. **L** The tumor volumes were measured every week. The FMD attenuated tumor growth in mice (*n* = 6), tumor weights, and tumor volumes by 4 weeks of cell transplantation. *P < 0.05, **P < 0.01, and ***P < 0.001.

To simulate fasting in PTC, we cultured TPC-1 and K1 cell lines in a fasting mimic medium and assessed cell proliferation and colony-forming units 24 and 48 h post fasting. Cell proliferation and colony formation was significantly lower after 24 h and 48 h of fasting (Fig. 1A, B). To better understand the effects of fasting on glucose metabolism, we performed a glucose uptake along with lactate production assay and found both were significantly decreased after 24 and 48 h of fasting (Fig. 1C). The extracellular acidification rate (ECAR), a common indicator for glycolysis, was observed to be significantly decreased under fasting conditions (Fig. 1D). Additionally, the oxygen consumption rate (OCR), which indicates mitochondrial respiration, was significantly increased under fasting conditions (Figs. 1E and S2A, B). We next assessed the levels of glycolytic enzymes such as 3-Phosphoinositide-dependent protein kinase 1 (PDK1), lactate dehydrogenase A (LDHA), hexokinase 2 (HK2), glucose transporter 1 (GLUT1) and glucose-6-phosphate isomerase (GPI). We observed a significant decrease in all the assessed enzymes by 48 h of fasting, thus indicating that fasting inhibited glycolysis in TPC-1 and K1 cells (Fig. 1F). However, TPC1 and K1 carried mutations such as RET/PTC1 rearrangement or BRAF^V600E^, and their effects in glycolysis is unknown in PTC [29, 30]. To eliminate the effect of mutations on glycolysis, TPC-1 cells were treated with SU11248, while K1 cells were treated with Vemurafenib before cultured in the fasting medium. Then the glucose uptake, lactate production, ECAR, OCR, and glycolytic enzymes were determined, the results indicated that BRAF^V600E^ and RET/PTC1 rearrangement in K1 and TPC-1 cells has very little promotion on glycolysis (Fig. S1A–F). Thus, fasting significantly inhibits glycolysis independent of genetic mutations in TPC-1 and K1 cells. Afterwards, to evaluate the effect of fasting on mitochondrial fusion and fission, relative mitochondrial ATP and ROS production was assessed, and it was evident that fasting increased mitochondrial ATP production while decreasing the ROS production (Fig. 1G). The mitochondrial membrane potential was then assessed using JC-1 dye, and we found that fasting significantly increased JC-1 mitochondrial aggregates, indicating an increase in membrane potential (Fig. 1H). Further, imaging indicated that mitochondria were less fragmented and more elongated with an increase in fasting (Fig. 1I). To understand the mitochondrial dynamics during fasting, we assessed the levels of mitochondrial fusion proteins mitofusin 1 (MFN1), MFN2, optic atrophy 1 (OPA1) and fission proteins dynamin-related protein 1 (DRP1), fission 1 (FIS1), mitochondrial fission factor (MFF) in cell models. We observed a significant increase in fusion proteins levels and decrease in the fission protein after fasting (Fig. 1J). To understand the role of fasting on PTC in vivo, we subcutaneously injected TPC-1 and K1 cells into BALB/c mice which were randomly separated into two groups, control and FMD group. After being monitored for four consequent weeks, mice in both two groups were sacrificed and the tumors were removed and assessed. During the course of the FMD procedure, we conducted measurements and collection of blood samples from the mice on the last day of the experiments. Subsequently, we examined the levels of fasting plasma glucose and insulin concentrations. These findings indicated that the mice in the FMD group experienced a reduction of around 10% of their body weight, which subsequently regained most of their body weight after refeeding (Fig. S3A). FMD demonstrated the capacity to decrease levels of fasting blood glucose and insulin concentrations (Fig. S3B, C). Moreover, among the FMD mice, we observed smaller tumors as early as 2 weeks post transplantation (Fig. 1K, L). We imaged and weighed the dissected tumors further and found that the FMD group had significantly smaller tumors than the control groups.

### PRKCQ-AS1, a downstream target of fasting, overexpression inhibits while knockdown promotes PTC cell proliferation in vitro and in vivo

**Fig. 2:**
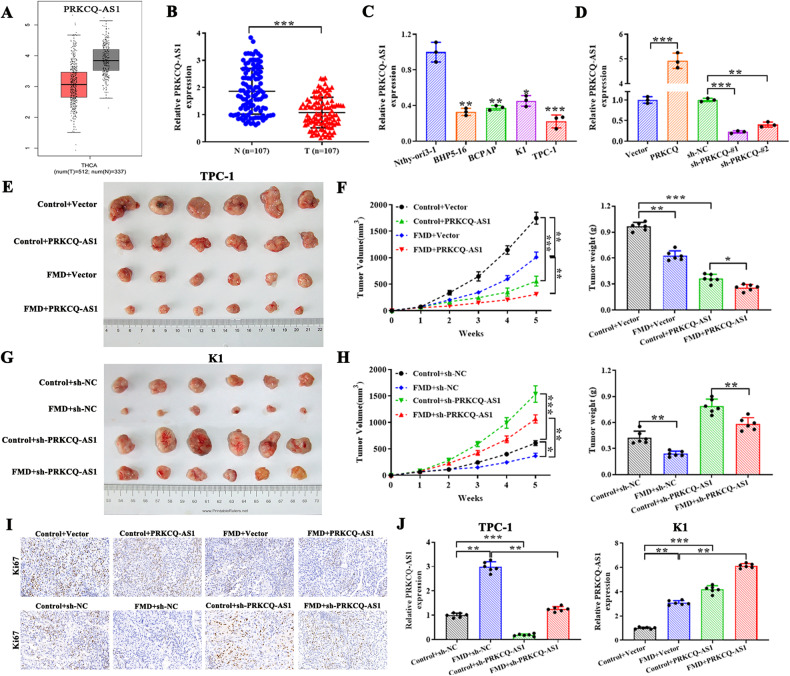
lncRNA PRKCQ-AS1, a downstream target of fasting, overexpression inhibits while knockdown promotes PTC cell proliferation in vitro and in vivo. **A** The expression of lncRNA PRKCQ-AS1 in THCA from the TCGA database. **B** Expression of lncRNA PRKCQ-AS1 was detected by qRT-PCR in PTC tissues (*n* = 107). **C** LncRNA expression of PRKCQ-AS1 was detected by qRT-PCR in PTC cell lines, compared to Nthy-ori3-1 normal cells. **D** LncRNA expression of PRKCQ-AS1 in K1 cells transfected with vector, PRKCQ-AS1, sh-NC, sh-PRKCQ-AS1-1# or sh-PRKCQ-AS1-2# was assessed by qRT-PCR. **E**, **F** Photograph of dissected tumors were shown. TPC-1 cells were transfected with Control+vector, control+PRKCQ-AS1, FMD+vector, and FMD + PRKCQ-AS1. Tumor volume and tumor weight in different groups were quantified and illustrated. **G**, **H** Photograph of dissected tumors. K1 cells were transfected with Control+sh-NC, control+sh-PRKCQ-AS1, FMD+sh-NC, and FMD+sh-PRKCQ-AS1. Tumor volume and tumor weight in different groups were quantified and illustrated. **I** Immunohistochemical staining of ki-67 positivity in tumors of different groups. Scale bar=50 µm. **J** The lncRNA expression of PRKCQ-AS1 was detected by qRT-PCR in tumors of different groups. *P < 0.05, **P < 0.01 and ***P < 0.001.

Two microarray datasets (GSE3467 and GSE3678) from the GEO database are obtained, which consist of a combined total of 16 PTC samples and 16 corresponding normal samples. We inferred that PRKCQ-AS1 is significantly downregulated in PTC tumors compared to corresponding normal samples (Fig. S4A, B). Subsequently, an evaluation on the TCGA-THCA dataset was conducted, revealing a notable decrease in the expression levels of PRKCQ-AS1 in PTC tissues compared to the control group (Fig. 2A). We then used qRT-PCR to examine the expression of PRKCQ-AS1 in PTC tissues and discovered that PTC tissues had significantly lower PRKCQ-AS1 levels than normal tissue samples (Fig. 2B). In vitro analysis revealed that PTC cell lines had significantly lower levels of PRKCQ-AS1 than normal cells (Fig. 2C). We further overexpressed or silenced PRKCQ-AS1 in TPC-1/K1cells and observed that sh-PRKCQ-AS1-#1 was much lower than sh-PRKCQ-AS1-#2, so sh-PRKCQ-AS1-#1 (namely sh-PRKCQ-AS1) were used for subsequent experiments (Fig. 2D). To elucidate PRKCQ-AS1’s role on PTC in vivo, we subcutaneously transplanted TPC-1 cells overexpressing PRKCQ-AS1 along with their respective controls into BALB/c nude mice. These mice were split into two random groups under FMD or normal diet (control group). After 5 weeks, the mice were sacrificed and tumors were dissected for further assessment (Fig. 2E). Interestingly, tumors in the control group were significantly larger than that of the PRKCQ-AS1 overexpression group. Further, FMD in combination with PRKCQ-AS1 overexpression significantly decreased the tumor size, indicating the importance of PRKCQ-AS1 (Fig. 2E, F). However, silencing of PRKCQ-AS1 significantly increased the tumor size (Fig. 2G, H). Immunostaining of the tumorous tissues indicated that overexpression of PRKCQ-AS1 decreased Ki-67 staining, which was further decreased in mice under FMD (Fig. 2I). Alternatively, silencing of PRKCQ-AS1 significantly increased Ki-67 staining which was decreased in mice under FMD (Fig. 2I). qRT-PCR confirmed that tumor overexpressing PRKCQ-AS1 had significantly higher levels of PRKCQ-AS1, which were further increased by FMD. Silencing of PRKCQ-AS1 significantly decreased PRKCQ-AS1 levels, which could be recovered slightly under FMD (Fig. 2J). These results clearly indicated that PRKCQ-AS1 could significantly decrease tumorigenesis in PTC which could be further augmented by FMD.

### PRKCQ-AS1 knockdown increases whereas PRKCQ-AS1 overexpression reduces cell cycle and migration/invasion under fasting mimic treatment

**Fig. 3:**
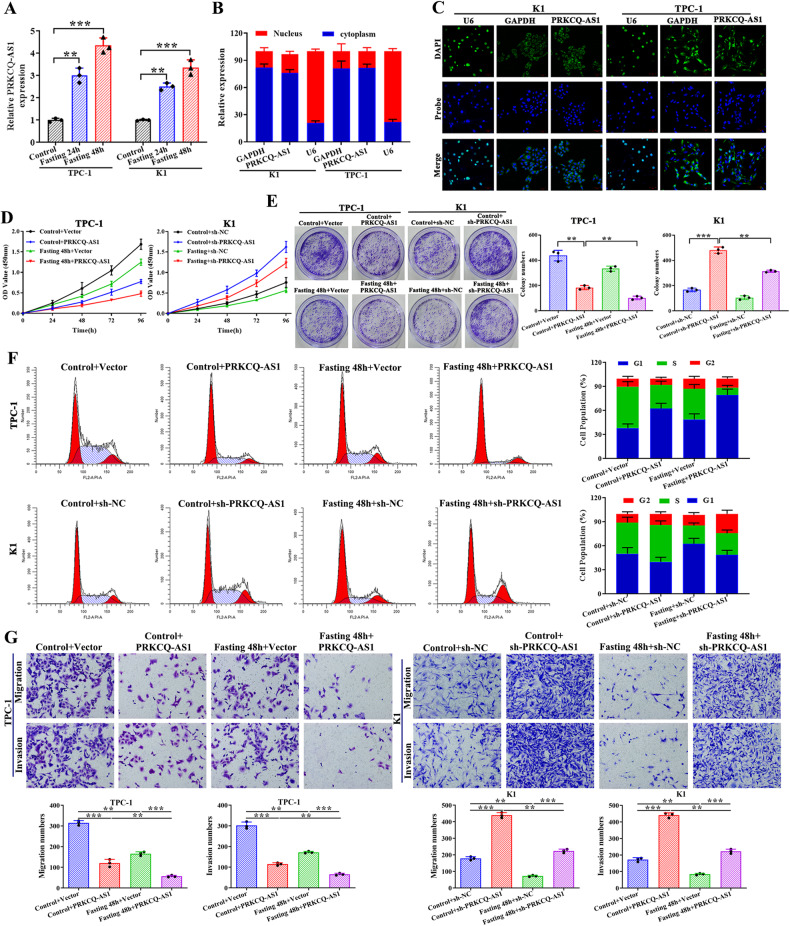
PRKCQ-AS1 knockdown increases whereas PRKCQ-AS1 overexpression reduces cell cycle and migration/invasion under fasting mimic treatment. **A** Quantitative RT-PCR analysis for PRKCQ-AS1 lncRNA expression levels in TPC-1 and K1 cells cultured in the fasting mimic medium. **B** Nuclear and cytoplasmic expression of PRKCQ-AS1 in TPC-1 and K1 cells (U6 was used as a nuclear control and GAPDH was used as a cytoplasmic control). **C** RNA FISH analysis of PRKCQ-AS1 in TPC-1 and K1 cells. **D**, **E** PRKCQ-AS1 overexpression/knockdown and fasting for 48 h inhibited TPC-1 and K1 cell proliferation, which was measured by CCK8 assay and colony formation assay. **F** The cell cycle distribution of PRKCQ-AS1 overexpressed cells after fasting for 48 h group was analyzed by flow cytometric analysis. **G** Migration/invasion capabilities of PRKCQ-AS1-overexpression TPC-1 cells and PRKCQ-AS1-knockdown K1 cells with fasting 48 h treatment were evaluated with Matrigel-coated Transwell assays. *P < 0.05, **P < 0.01 and ***P < 0.001.

Further, we assessed PTC cell proliferation, cell cycle, and migration/invasion in PRKCQ-AS1-overexpressing TPC-1 cells and PRKCQ-AS1-silencing K1 cells under fasting mimic medium. TPC-1 and K1 cell culture under fasting conditions significantly increased PRKCQ-AS1 expression levels (Fig. 3A). These results clearly indicated that fasting could increase PRKCQ-AS1expression levels in TPC-1 and K1 cells, and PRKCQ-AS1 is a downstream target of fasting. In both TPC-1 and K1 cells, the sub-cellular expression profile revealed that PRKCQ-AS1 transcripts were highly expressed in the cytoplasm rather than the nucleus (Fig. 3B). These results were further confirmed using FISH analysis (Fig. 3C). Moreover, when PRKCQ-AS1 was overexpressed in combination with a 48-h fasting, it resulted in a significant decrease in both proliferation and colony formation (Fig. 3D, E). Similarly, silencing of PRKCQ-AS1 increased proliferation and colony formation units significantly. Cell cycle analysis revealed that overexpression of PRKCQ-AS1 shifted a significant population of cells into G1 phase from S phase, which was higher along with fasting, whereas silencing of PRKCQ-AS1 along with fasting showed a high population of cells in S phase and less in the G1 phase (Fig. 3F). Additionally, we observed that overexpression of PRKCQ-AS1 decreased TPC-1 cell migration/invasion, and the addition of fasting mimic treatment could aggravate these effects (Fig. 3G). Whereas, knockdown of PRKCQ-AS1 increased K1 cell migration/invasion, the addition of fasting mimic treatment could alleviate these effects (Fig. 3G). These results indicated that PRKCQ-AS1 knockdown increases whereas PRKCQ-AS1 overexpression reduces migration/invasion under fasting mimic treatment.

### PRKCQ-AS1 interacts with IGF2BPs in PTC

**Fig. 4:**
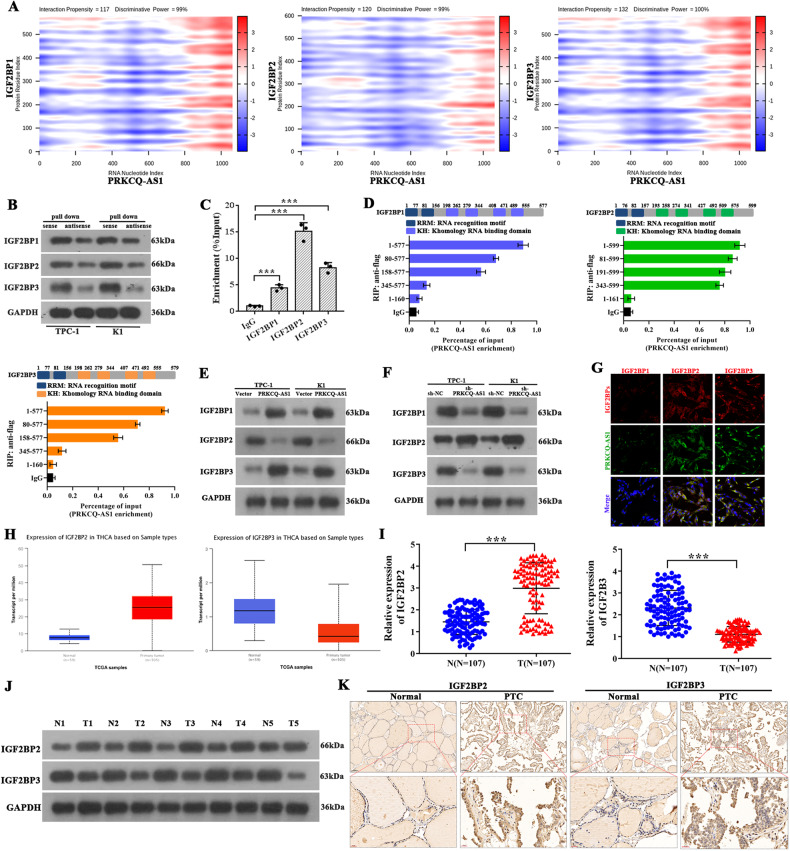
PRKCQ-AS1 interacts with IGF2BPs in PTC. **A** CatRAPID prediction result of lncRNA PRKCQ-AS1 and IGF2BPs shown as a heat-map. The X- and Y-axes represent the indexes of the RNA and protein sequences, respectively. The colors of the heat-map indicate the interaction score of the individual amino acid and nucleotide pairs. **B** Immunoblotting for the specific associations of IGF2BP1, IGF2BP2, or IGF2BP3 with biotinylated-PRKCQ-AS1 from three independent streptavidin RNA pull-down assays in TPC-1 and K1 cells. **C** Analysis for PRKCQ-AS1 enrichment. RIP assay was performed using IGF2BP antibodies in TPC-1 cells. **D** Deletion mapping for the domains of IGF2BP1, IGF2BP2, and IGF2BP3 that bind to PRKCQ-AS1. RIP analysis for PRKCQ-AS1 enrichment in TPC-1 cells transiently transfected with plasmids containing the indicated FLAG-tagged or HA-tagged full length or truncated constructs. **E**, **F** Protein levels of IGF2BPs in TPC-1 and K1 cells with PRKCQ-AS1 overexpression (**E**) or knockdown (**F**), respectively. **G** Co-localization of PRKCQ-AS1 with IGF2BPs, in TPC-1 cells. Scale bar = 50 μm. **H** The expression of IGF2BP2 and IGF2BP3 in THCA from TCGA database were shown. **I** mRNA expression of IGF2BP2 and IGF2BP3 was determined by qRT-PCR in PTC tissues (*n* = 107). **J** Protein expression of IGF2BP2 and IGF2BP3 were determined by western blot in PTC tissues (*n* = 5). **K** The expression of IGF2BP2 and IGF2BP3 in PTC tissues and adjacent normal tissues were examined by immunohistochemistry. *P < 0.05, **P < 0.01 and ***P < 0.001.

To clarify the mechanism of PRKCQ-AS1 in PTC, we analyzed its downstream regulators using various bioinformatic tools. Using catRAPID omics tool, we assessed PRKCQ-AS1’s interactions with other proteins. The nucleotide sequence of lncRNA PRKCQ-AS1 was compared to the human proteome to identify potential RNA-protein interactions. Interestingly, we found IGF2BP1, IGF2BP2, and IGF2BP3 to strongly interact with PRKCQ-AS1. IGF2BPs have been previously recognized as an m^6^A reader whose methylation is critical in tumorigenesis [31]. Further, we created heat maps of nucleotide and amino acid interactions between PRKCQ-AS1 and IGF2BPs using catRAPID graphics tool (Fig. 4A). Interestingly, we observed that IGF2BP3 interacted with PRKCQ-AS1 at the highest propensity, followed by IGFBP2 and IGFBP1. We further confirmed these using TPC-1 and K1 cell derived RNA-pull down assay followed by western blotting with IGF2BP antibodies. IGF2BP1, IGF2BP2 and IGF2BP3 strongly precipitated with sense lncRNA PRKCQ-AS1 than that of antisense strand (Fig. 4B). RIP assays for FLAG-tagged full-length and truncated IGF2BP1 and 3 showed that the deletion of K homology RNA-binding domains (KH1 and KH2) significantly abolished their association with PRKCQ-AS1. Additionally, the deletion of KH3 and KH4 of IGF2BP2 exhibited similar effects, indicating PRKCQ-AS1 binds to these regions (Fig. 4C, D). We silenced or overexpressed PRKCQ-AS1 in TPC-1 and K1 cells and tested the mRNA and protein levels of IGF2BPs. We observed that indeed overexpression of PRKCQ-AS1 significantly increased IGF2BP1 and IGF2BP3 while decreasing IGF2BP2 levels, whereas silencing caused a reverse effect (Fig. 4E, F). Immunofluorescence assays clearly showed the strong co-localization of PRKCQ-AS1 with IGF2BP2 and IGF2BP3, but IGF2BP1 is very lower (Fig. 4G). The TCGA database was used to identify expression patterns of IGF2BPs and showed that in PTC tumor samples IGF2BP3 were expressed in low levels while IGFBP2 was highly expressed, IGF2BP1 has barely any difference, compared to normal samples (Figs. 4H and S5A). We next assessed the levels of IGF2BPs by qRT-PCR western blot and immunohistochemical staining, and observed that IGF2BP3 has significantly lower levels while IGF2BP2 has significantly higher levels than IGF2BP1 in PTC tissues, compared to normal tissues (Figs. 4I–K and S5B).

### PRKCQ-AS1 regulates glycolysis and mitochondrial dysfunction of PTC through targeting IGF2BPs in vitro

**Fig. 5:**
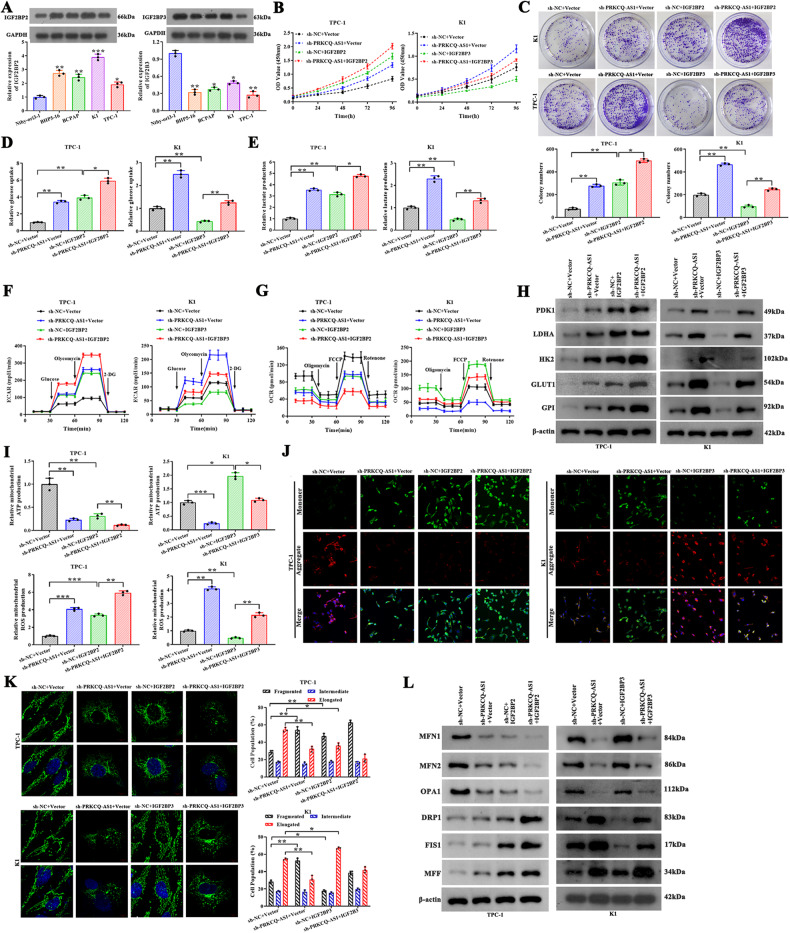
PRKCQ-AS1 regulates glycolysis and mitochondrial dysfunction of PTC through targeting IGF2BPs in vitro. **A** The mRNA and protein expressions of IGF2BPs were detected by qRT-PCR and western blot in PTC cells. **B**–**L** PTC-1 cells were co-transfecred with sh-NC/sh-PRKCQ-AS1 and Vector/IGF2BP2; while K1 cells were co-transfecred with sh-NC/sh-PRKCQ-AS1 and Vector/IGF2BP3. **B**, **C** Cell proliferation was determined by CCK8 assay and colony formation assay in PTC-1 and K1 cells. **D**–**G** Glucose uptake, lactate production, ECAR and OCR were examined in PTC-1 and K1 cells. **H** Glycolysis associated proteins (PDK1, LDHA, HK2, GLUT1, and GPI) were determined by western blot. **I** Mitochondrial ROS and ATP production were observed. **J** JC-1 staining was used to observe mitochondrial membrane potential. Scale bar, 50 μm. **K** The proportion of cells with elongated, intermediate and fragmented mitochondrial was quantified. Scale bar, 10 μm. **L** Western blot and qRT-PCR analysis for protein expression levels of MFN1, MFN2, OPA1, DRP1, FIS1, and MFF. *P < 0.05, **P < 0.01, and ***P < 0.001.

Assessment of IGF2BPs levels in PTC cell lines indicated that TPC-1 had the least level of IGF2BP3 while K1 had the highest IGF2BP2 and there is no difference in IGF2BP1 (Figs. 5A and S5C). So, we focused on IGF2BP2 and IGF2BP3 for subsequent experiments. Moreover, the transfection efficiency experiments confirmed that IGF2BP2/3 overexpression has higher IGF2BP2/3 expression levels, IGF2BP2/3 knockdown has lower expression levels (Fig. S6A). Cell population analysis indicated that overexpression of IGF2BP2 moved a huge population of cells to S phase, whereas overexpression of IGF2BP3 moved cells to G1 phase. Silencing of IGF2BP2/3 resulted in the opposite (Fig. S6B). Further to understand the dynamic interactions between PRKCQ-AS1 and IGF2BP2/3, we silenced PRKCQ-AS1 and overexpressed either IGF2BP2 or IGF2BP3 in PTC-1 and K1 cells. In IGF2BP2 overexpressing-PTC-1 cells, cell proliferation, colony formation unit, glucose uptake, lactate production, ECAR, glycolytic enzyme levels and ROS production increased, whereas OCR and mitochondrial ATP production significantly decreased, which was further augmented by PRKCQ-AS1 knockdown (Figs. 5B–I and S2C, left). Additionally, in IGF2BP3 overexpressing-K1 cells, all of these indicators were opposite, and reversed by PRKCQ-AS1 knockdown (Figs. 5B–I and S2C, right). Overexpression of IGF2BP2 significantly increased JC-1 mitochondrial monomers with fragmented mitochondrial structures and fission protein level in PTC-1 cells, while overexpression of IGF2BP3 increased mitochondrial aggregates with elongated mitochondrial structures and fusion protein level in K1 cells. However, this increase in aggregates, elongated structures, and fusion protein level were reversed by the silencing of PRKCQ-AS1 (Fig. 5J–L). All these results indicated that silencing of PRKCQ-AS1 enhanced the effects of IGF2BP2 overexpressing and reversed the effects of IGF2BP3 overexpressing confirmed that indeed PRKCQ-AS1 regulates glycolysis and mitochondrial dysfunction of PTC through targeting IGF2BPs.

### PRKCQ-AS1 facilitates PRMT7 expression via interacting with IGF2BPs

**Fig. 6:**
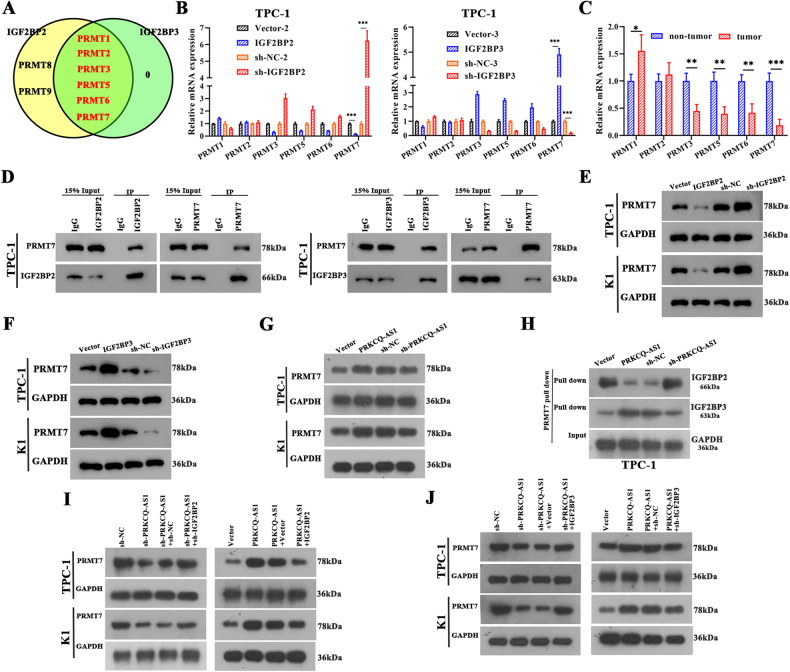
PRKCQ-AS1 facilitates PRMT7 expression via interacting with IGF2BPs. **A** Representation of overlapping IGF2BP2 and IGF2BP3 which potentially bind with PRMT family of RNA, as identified by Starbase 3.0. **B** The mRNA expression of PRMTs was examined by qRT-PCR in TPC-1 cells transfected with vector and IGF2BP2/3 or sh-NC and sh-IGF2BP2/3. **C** The mRNA expression of PRMTs was examined by qRT-PCR in PTC tissues. **D** TPC-1 cell lysates were subjected to immunoprecipitation with control IgG, anti-IGF2BP2, anti-IGF2BP3 or anti-PRMT7 antibody. The immunoprecipitates were then blotted. **E**, **F** Western blot analysis for PRMT7 protein levels in TPC-1 and K1 cells, which was transfected with vector/IGF2BP2/3 or sh-NC/sh-IGF2BP2/3. **G** Western blot analysis for PRMT7 protein levels in TPC-1 and K1 cells, which were transfected with vector/PRKCQ-AS1 or sh-NC/sh-PRKCQ-AS1. **H** Immunoblot analysis showed the specific association of IGF2BP2/3 with PRMT7 mRNA in TPC-1 cells with PRKCQ-AS1 overexpression or knockdown. **I** TPC-1 cells were transfected with sh-NC, sh-PRKCQ-AS1, sh-PRKCQ-AS1+sh-NC or sh-PRKCQ-AS1+sh-IGF2BP2; K1 cells were transfected with vector, PRKCQ-AS1, PRKCQ-AS1+vector or PRKCQ-AS1 + IGF2BP2. Western blot was used to analyze PRMT7 protein expression. **J** TPC-1 cells were transfected with sh-NC, sh-PRKCQ-AS1, sh-PRKCQ-AS1+vector or sh-PRKCQ-AS1 + IGF2BP3; K1 cells were transfected with vector, PRKCQ-AS1, PRKCQ-AS1+sh-NC or PRKCQ-AS1+sh-IGF2BP3. Western blot was used to analyze PRMT7 protein expression. *P < 0.05, **P < 0.01 and ***P < 0.001.

To further understand the mechanism of regulating glycolysis and mitochondrial function, we assessed potential targets of IGF2BPs. Interestingly, we found a family of protein arginine methyltransferases (PRMTs) to be potential targets of IGF2BPs. It has been reported that IGFBP2 stabilizes PRMT6 mRNA [32], IGFBP1/3 indirectly enhance the protein stability of PRMT5 [33], and there is also a regulatory relationship between PRMT3 and IGFBP1 [34]. Starbase 3.0 (https://starbase.sysu.edu.cn/) identified that IGF2BP2 and IGF2BP3 bind to the protein PRMT1,2,3,5,6,7 (Fig. 6A). Further, when we overexpressed IGF2BP2, PRMT7 mRNA expression level decreased significantly, and silencing of IGF2BP2 did the opposite. Alternatively, overexpression of IGF2BP3 significantly increased PRMT7 level, whereas silencing of IGF2BP3 significantly decreased it (Fig. 6B). Assessment of mRNA levels showed that PRMT7 were much lower in tumor samples than other PRMTs (Fig. 6C). Furthermore, Co-IP analysis indicated that PRMT7 and IGF2BP2/3 interacted strongly with each other (Fig. 6D). Additionally, we assessed the protein levels of PRMT7 in the presence or absence of IGF2BP2/IGF2BP3. As expected, lack of IGF2BP2 increased PRMT7 levels, whereas overexpression of IGF2BP2 decreased it. The effects of IGF2BP3 overexpression and silencing were opposite (Fig. 6E, F). Additionally, PRKCQ-AS1 increased PRMT7 levels, whereas silencing of PRKCQ-AS1 significantly decreased its levels (Fig. 6G). We further confirmed the association between PRMT7 mRNA and IGF2BP2/3 by performing RNA pull down assays. When cells overexpressing PRKCQ-AS1, PRMT7 pull down precipitates had very low levels of IGF2BP2 but high levels of IGF2BP3 (Fig. 6H). However, when PRKCQ-AS1 was silenced, pull down precipitates had the opposite results. Next, we silenced PRKCQ-AS1 along with IGF2BP2/IGF2BP3 to assess PRMT7 levels (Fig. 6I, J). Interestingly, PRKCQ-AS1 knockdown decreased PRMT7 expression, which was abolished by the addition of IGF2BP2 knockdown; conversely, PRKCQ-AS1 overexpression promoted PRMT7 expression, which was alleviated by the addition of IGF2BP2. However, when we silenced IGF2BP3 along with PRKCQ-AS1, PRMT7 levels has the opposite effects. So, these results clearly indicated that PRKCQ-AS1 regulates the expression of PRMT7 via IGF2BPs.

### PRMT7 is required for PRKCQ-AS1’s effect on regulation of PTC cell proliferation and cell cycle

**Fig. 7:**
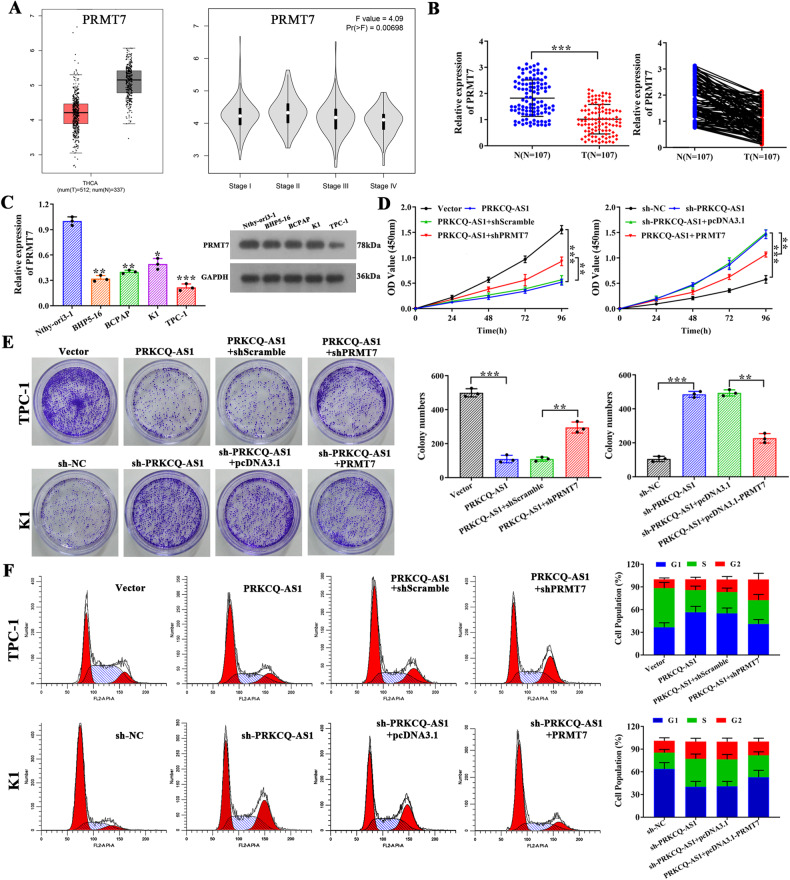
PRMT7 is required for PRKCQ-AS1’s effect on regulation of PTC cell proliferation and cell cycle. **A** Expression and tumor stage of PRMT7 in THCA in TCGA database were shown. **B** The mRNA expression of PRMT7 was detected by qRT-PCR in PTC tissues (*n* = 107). **C** The mRNA and protein expression of PRMT7 were detected by qRT-PCR and western blot in PTC cell lines. **D**–**F** TPC-1 cells were transfected with vector, PRKCQ-AS1, PRKCQ-AS1+shScramble, or PRKCQ-AS1+shPRMT7 and K1 cells were transfected with sh-NC, sh-PRKCQ-AS1, sh-PRKCQ-AS1+pcDNA3.1, or sh-PRKCQ-AS1 + PRMT7. **D** Cell proliferation was determined by CCK8 assay. **E** Colony formation assay were observed. **F** The cell cycle distribution was analyzed by flow cytometry analysis. *P < 0.05, **P < 0.01 and ***P < 0.001.

Further, PRMT7 levels were assessed in the THCA database. Evidentially, PRMT7 levels were significantly lower in PTC patients, but there was no significant difference between stages of PTC. (Fig. 7A). Additionally, we assessed the mRNA expression level using qRT-PCR and confirmed significantly lower levels of expression in tumor samples (Fig. 7B). Assessment of PRMT7 levels in PTC cell lines indicated significantly low mRNA expression levels in all the PTC cells (Fig. 7C). Furthermore, the overexpression and knockdown efficiency of PRMT7 were confirmed both in mRNA and protein levels by qRT-PCR and western blot in K1 and TPC-1 cells **(**Fig. S7A**)**. Silencing of PRMT7 reversed the effects of PRKCQ-AS1 overexpression on cell proliferation and colony forming of TPC-1 cells, and overexpression of PRMT7 reversed silencing of PRKCQ-AS1 similarly (Fig. 7D, E). Cell cycle analysis showed the effect of PRKCQ-AS1 overexpressed on cell population in G1 phase was reversed by PRMT7 silencing, additionally, similar effect occurs between PRKCQ-AS1 silencing and PRMT7 overexpressing (Fig. 7F). These results indicated that indeed PRMT7 is essential for PRKCQ-AS1’s effect on PTC.

### PRKCQ-AS1 regulates glycolysis and mitochondrial dysfunction through IGF2BPs/PRMT7 signaling pathway in vitro

**Fig. 8:**
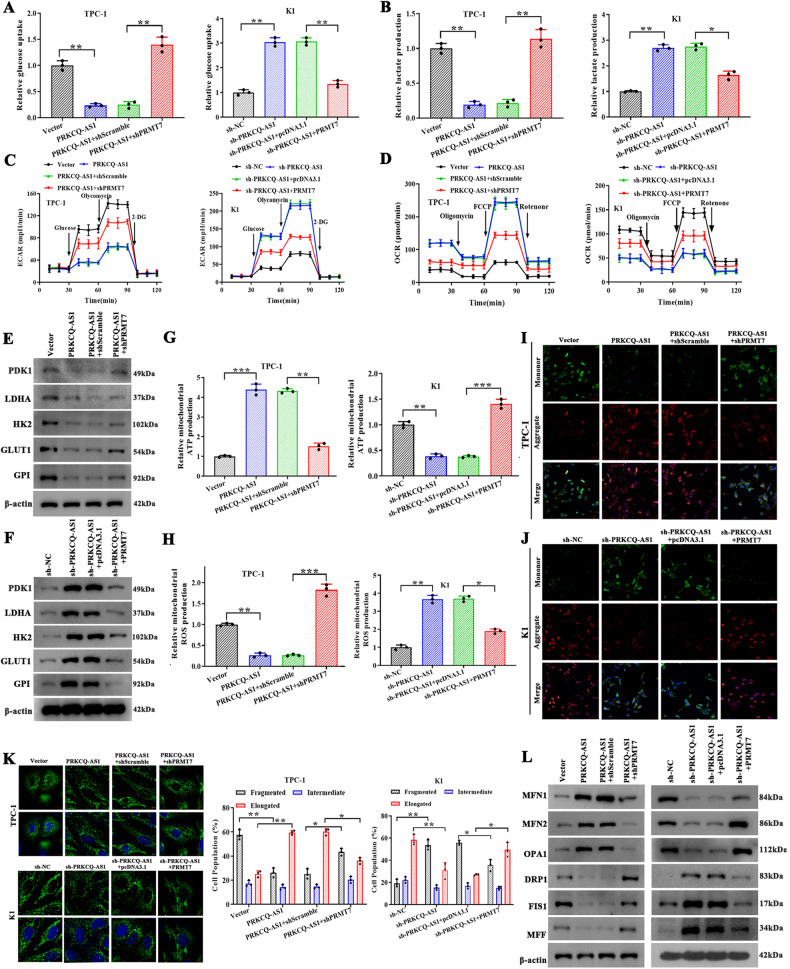
PRKCQ-AS1 regulates glycolysis and mitochondrial dysfunction through IGF2BPs/PRMT7 signaling pathway in PTC cells. **A**–**F** TPC-1 cells were transfected with vector, PRKCQ-AS1, PRKCQ-AS1+shScramble, or PRKCQ-AS1+shPRMT7; K1 cells were transfected with vector, PRKCQ-AS1, PRKCQ-AS1 +shScramble, or PRKCQ-AS1+shPRMT7. **A**–**D** Glucose uptake, lactate production, ECAR and OCR were examined. **E**, **F** Glycolytic related proteins (PDK1, LDHA, HK2, GLUT1 and GPI) were determined by western blot (upper: TPC-1; lower: K1). **G**, **H** Mitochondrial ROS and ATP production were observed. **I**–**J** JC-1 staining was used to observe mitochondrial membrane potential. Scale bars, 50 μm. **K** The proportion of cells with elongated, intermediate and fragmented mitochondrial was quantified. Scale bars, 10 μm. **L** Western blot and qRT-PCR analysis for protein and mRNA expression levels of MFN1, MFN2, OPA1, DRP1, FIS1, and MFF (Left: TPC-1; Right: K1). *P < 0.05, **P < 0.01, and ***P < 0.001.

To understand further the effect of PRMT7 on glycolysis, we assessed glucose uptake, lactate production, ECAR, OCR, expression of glycolysis proteins, mitochondrial ATP, and ROS production in TPC-1 cells overexpressed or silenced for PRMT7 along with PRKCQ-AS1 (Figs. 8A–H and S2D). Additionally, we also assessed mitochondrial membrane potential, mitochondrial morphology, and expression of fusion and fission proteins in the above cells to understand the effect of PRMT7 on mitochondrial dysfunction (Fig. 8I–L). Results of these assays indicated the effect of overexpression of PRKCQ-AS1 that reduces glycolysis and mitochondrial dysfunction was reversed by silencing of PRMT7, and the effect of silencing of PRKCQ-AS1 that increases glycolysis and mitochondrial dysfunction was also reversed by overexpression of PRMT7. Furthermore, we assessed the effect of fasting on IGF2BP2/3 and PRMT7 mRNA and protein expression levels in TPC-1 and K1 cells under control and fasting medium. The results indicated that fasting decreased IGF2BP2 expression, which is exacerbated by PRKCQ-AS1 overexpression and alleviated by PRKCQ-AS1 knockdown; meanwhile, fasting promotes PRMT7 expression, while was promoted by PRKCQ-AS1 overexpression and inhibited by PRKCQ-AS1 knockdown in TPC-1 and K1 cells (Fig. S8A, B). On the contrary, fasting increases IGF2BP3 expression, and these are promoted by PRKCQ-AS1 overexpression and inhibited by PRKCQ-AS1 knockdown; and that, fasting promotes PRMT7 expression, while was promoted by PRKCQ-AS1 overexpression and inhibited by PRKCQ-AS1 knockdown in TPC-1 and K1 cells (Fig. S8C, D). In conclusion, these results clearly indicated that fasting regulates glycolysis and mitochondrial dysfunction via the PRKCQ-AS1/IGF2BPs/PRMT7 signaling pathway in PTC.

## Discussion

Cancer cells have immense energy needs for their increased proliferation and metastasis, which is typically met by increasing glycolysis and decreasing mitochondrial oxidative phosphorylation. This transformation in energy needs is termed as the Warburg effect [35, 36]. Relative literature reported that TPC1 and K1 carried mutations such as RET/PTC1 rearrangement or BRAF^V600E^, and their effects in glycolysis are unknown in PTC [29, 30]. Numerous studies indicated that BRAF(V600E) mutant melanoma is one of the most glycolytic cancers. In BRAF(V600E) melanoma cells, activation of BRAF upregulates glycolysis and suppresses oxidative phosphorylation (OXPHOS) [37–39]. In papillary thyroid carcinoma, BARF^V600E^ is one of the most common genetic mutations associated with 40–80% PTC pathogenesis. And that, BRAF mutations activate the MAPK pathway, resulting in increases in cell proliferation, dedifferentiation, and apoptosis [40, 41]. Nevertheless, there are no reports on glycolysis through MARK pathway in PTC. Up to now, Gao et al. constructed BRAF^V600E^-overexpressing and BRAF^V600E^-knockdown thyroid cell lines for glycolysis experiments, the results indicated that the overexpression of BRAF^V600E^ enhanced aerobic glycolysis [29]. Couto et al. introduced that human thyroid cancer-derived cell lines [harboring RET/PTC or BRAF)] promoted STAT3 activation; STAT3 knockdown promoted aerobic glycolysis through increasing in glucose consumption, lactate production, and HIF1α [42]. However, the effect in details and the underlying mechanism of genes mutations on glycolysis in PTC has not been reported. In our study, TPC-1 cells were treated with SU11248, while K1 cells were treated with Vemurafenib before cultured in the fasting medium. The results indicated that BRAF^V600E^ and RET/PTC rearrangement has very little promotion on glycolysis (Fig. S1). Through the little promotion of gene mutations on glycolysis did not affect the inhibition of fasting on glycolysis in PTC, there are some discrepancies between these results and the reports. So, the effect of K1 cells harboring the BRAF^V600E^ mutation, or TPC-1 harboring the RET/PTC rearrangement on glycolysis needs to be further explored in depth, the molecular regulatory mechanisms need to be further investigated.

Fasting diets have been proven effective against many types of cancers [43, 44]. FMD has previously been identified to reduce this reliance on glycolysis, forcing cancer cells to reduce their proliferation and, as a result, the progression of tumorigenesis [11]. However, fasting in tumor progression and treatment has not yet been studied and reported in clinical study, including PTC, which is a clinical limitation of this manuscript. In this study, we observed a significant decrease in proteins involved in glycolysis under FMD. Additionally, we observed an increase mitochondrial fusion proteins and a decrease in fission proteins. Mitochondrial dynamics is critical for meeting cellular energy demands; mitochondrial fusion is frequently associated with increased ATP production, decreased ROS production, and impaired oxidative phosphorylation. Upregulation of fission proteins, on the other hand, is associated with increased ROS production and restricted oxidative phosphorylation [45]. Upregulation of the mitochondrial fission protein Drp1 has been linked to tumorigenesis in prostate cancer studies [46], whereas downregulation of fusion protein Mfn1 has been linked to tumorigenesis in hepatocellular carcinoma studies [47]. In our study, fasting increased fusion proteins such as Mfn1, Mfn2, and Opa1, as well as increased ATP production and decreased ROS production.

Rodent studies have shown that FMD reduces inflammation and cancer incidence while improving overall health and cognitive performance [48]. We wanted to further understand the molecular causes for FMD’s effect on PTC. We found a significant increase in lncRNA PRKCQ-AS1 after fasting, and low levels of lncRNA PRKCQ-AS1 have been linked to a poor prognosis and survival in PTC patients. Interestingly, there have only been two studies on lncRNA-PRKCQ-AS1 in colorectal cancer (CRC), with no studies on PTC. In CRC, both the studies indicated that high expression of PRKCQ-AS1 led to poor prognosis and survival among CRC patients [15, 17]. Our study indicated the opposite i.e., high expression led to increased survival among PTC patients. This variation in effects based on cancer type is not unexpected, as it has been observed with other non-coding RNA and cancer types. In studies on the lncRNA BANCR, knockdown of BANCR significantly reduced tumor growth in BALB/c mouse CRC models [49]. On the other hand, overexpression of BANCR reduced tumor growth in BALB/c PTC models [31]. Such differences between cancers suggest a potential need for additional functional studies in other models to better understand the role of the lncRNA. In both the in vitro and in vivo models used in this study, we observed a clear increase in lncRNA PRKCQ-AS1 under FMD led to a decrease in PTC cell proliferation and decrease in tumor size.

LncRNAs are now well accepted as molecules that interact with either RNA, DNA, chromatin, or proteins and thus functionally regulate their activity [50]. In this study, we observed that PRKCQ-AS1 binds strongly to IGF2BPs which we systematically illustrated to in turn regulate glycolysis and mitochondrial function in PTC. We used bioinformatics tools such as CatRAPID to further understand the molecular etiology of PRKCQ-AS1’s role in PTC and discovered that the lncRNA PRKCQ-AS1 strongly binds and regulates the expression levels of RNA-binding proteins IGF2BPs. IGF2BP1 and 3, which share 75% sequence homology, are significantly downregulated in the absence of PRKCQ-AS1, whereas IGF2BP2 is highly upregulated in the absence of PRKCQ-AS1. However, the role of IGF2BPs in cancer prognosis is equally controversial. In gastrointestinal cancer, for example, increased IGF2BP3 expression has been linked to a poor prognosis, increased cancer cell proliferation, and metastasis [51]. However, in ovarian cancer IGF2BP3 expression has been associated with increased survival [52]. In this study, we found that PRKCQ-AS1 binds to IGF2BP1 and 3 via the RNA-binding protein’s KH1 and 2 domains, whereas it binds to IGF2BP2 via the protein’s KH3 and 4 domains. Indeed, the contradictory regulation of lncRNA may be due to the fact that binding to the KH1 and 2 domains makes the RNA-protein complexes stronger and more stable than binding to the other domains [18].

Furthermore, we discovered that IGF2BPs do indeed regulate tumorigenesis by binding to and regulating PRMT7. Co-immunoprecipitation studies indicated that both IGF2BP2 and 3 binds to and regulates the expression of PRMT7. Importantly, we identified that increased levels of IGF2BP2 significantly correlated with decreased PRMT7 levels and increased tumorigenesis. However, increased IGF2BP3 levels significantly increased PRMT7 levels and decreased PTC tumorigenesis. However, relatively less is known about its role in cancer. Two studies on breast cancer and lung cancer had associated increased PRMT7 levels to poor prognosis and survival [27, 28]. However, till date no study has assessed its role and expression in PTC.

PRMT7 is a member of a protein family that is essential for the post-transcriptional modification of histones via arginine methylation [53]. As global epigenetic changes through modification of methylation patterns play key roles in cancer development and progression, PRMT7’s role is evident and clear in PTC [54]. Interestingly, when compared to normal thyroid tissue samples, proteins in PTC tissues are found to be globally hypomethylated [55]. More functional studies linking histone methylation, PTC, and PRMT7 are needed to confirm the epigenetic relationships among them. This is, however, the first study to contribute to the identification of fasting and lncRNA-PRKCQ-AS1, to assess the prognosis of PTC. Furthermore, we identified the role of PRKCQ-AS1/IGF2BPs/PRMT7 in PTC models with FMD, shedding light not only on the molecular functionality of FMD but also allowing identification of potential PTC treatment targets.

## Materials and methods

### Cell culture and treatment

Cells lines Nthy-ori3-1, BHP5-16, BCPAP, K1, and TPC-1 used in this study were purchased from the Shanghai Cell Bank (Type Culture Collection Committee, Chinese Academy of Sciences, Beijing, China). Cells were cultured in a Dulbecco’s Modified Eagle’s Medium (DMEM, Gibco), supplemented with 10% fetal bovine serum (FBS, Gibco, Grand Island, NY, USA) and 1% penicillin/streptomycin at 37 °C in a CO_2_ incubator. The fasting mimic medium comprised of glucose-free DMEM supplemented with 0.5 g/L glucose and 1% FBS; fasting was mimicked by incubating cells in this medium for 24 h or 48 h. Before fasting medium, TPC-1 cells were treated with SU11248 (1 μM, 48 h); while K1 cells were treated with Vemurafenib, (1 μM, 48 h). Vemurafenib, a BRAF^V600E^ selective inhibitors, was purchased from Selleckchem (Houston, TX, USA). SU11248, a highly effective tyrosine inhibitor of the RET/PTC1 oncogenic kinase, was obtained from Pfizer, Inc.

### Clinical samples

PTC tissue and adjacent thyroid tissues were collected from 107 patients from the Shanghai Tenth People’s Hospital affiliated with Shanghai Tongji University and approved by the Research Ethics Committee of the Shanghai Tenth People’s Hospital (Shanghai, China). Patient studies were conducted in accordance with the Declaration of Helsinki. Patients did not receive local or systemic treatment prior to surgery. Written informed consent for research purposes was provided by all patients. All samples were processed as needed, i.e., frozen at −80 °C for RNA extraction and samples were embedded and stored for immunohistochemical analysis. The use of these samples was approved by ethics committee of Shanghai Tenth People’s hospital. Written informed consent was obtained from all the patients involved in this study.

### CCK 8 assay

Cells were initially seeded at 1500 cells/ well intensity onto a 96-well plate. After 24 h, CCK 8 assay was performed based on manufacturer’s instructions (CCK8; Dojindo Laboratories, Japan). Briefly, the cells were incubated 10 µl CCK8 solution and incubated for 4 h in incubator and the absorbance was measured at 450 nm using a microplate reader.

### Colony formation assay

Cells were seeded at a density of 1500 cells/well onto a 6-well plate. The cells were continued to be cultured till visible colonies were formed in the presence of complete growth medium. Cell colonies were finally fixed with methanol and stained with 0.1% crystal violet (Sigma, St. Louis, Mo) and counted.

### RNA isolation and qRT-PCR

RNA isolation of the samples was performed using mRNeasy mini kit (Qiagen) based on the manufacturer’s instructions. Further, the RNA isolated were reverse transcribed into complementary DNA with M-MLV Reverse Transcriptase Kit (Promega, Madison, WI, USA). qRT-PCR was performed using SYBR green RT-PCR kit (ThermoFisher, Waltham, MA, USA) with the primers used in this study are provided in the Table S1.

### Protein isolation and western blot

Cells and tissue samples were lysed using RIPA lysis buffer containing protease and phosphatase inhibitors. A total of 20 µg protein sample were loaded onto each well and resolved in a SDS gel. The samples were further transferred to a nitrocellulose membrane and the membrane were incubated with the below mentioned primary antibodies (IGF2BP1 (ab184305, Abcam); IGF2BP2 (ab124930, Abcam); IGF2BP3 (ab179807, Abcam); PRMT7 (ab181214, Abcam); MFN1 (ab129154, Abcam); MFN2 (ab205236, Abcam); OPA1 (ab157457, Abcam); DRP1 (ab184247, Abcam); FIS1 (ab156865, Abcam); MFF (ab129075, Abcam); PDK1 (ab202468, Abcam); LDHA (ab52488, Abcam); HK2 (ab227198, Abcam); GLUT1 (ab115730, Abcam); GPI (ab210753, Abcam); GAPDH (ab8245, Abcam); β-actin (ab8227, Abcam) after blocking with 5% skim milk. Finally, the membrane was incubated overnight with corresponding secondary antibodies before visualization using a chemiluminescence system (ECL).

### OCR and ECAR

Oxygen consumptive rate (OCR) and extracellular acidification rate (ECAR) in TPC-1 and K1 cell lines were determined using a Seahorse Bioscience XF96 Extracellular Flux Analyzer (Seahorse Bioscience, USA). Briefly, 20,000 cells were seeded 96 plate/well and incubated overnight. Cells were then incubated for 1 h at 37 °C with glucose (100 mM), oligomycin (10 μM), and 2-deoxy-glucose (2DG, 1 M). ECAR and OCR levels were then measured to determine glycolytic capacity.

### Glycolysis analysis

Glucose uptake analysis and lactate production analysis was performed using commercially available kits (Sigma-Aldrich, St. Louis, MO, USA), based on the manufacturer’s protocol. Briefly, 20,000 cells were seeded onto a 96 plate/well and incubated for 24 h before assessing the glucose uptake and lactate production.

### Cell cycle analysis

Cell cycle analysis were performed using propidium iodide staining (PI, Signal way Antibody, #CA002) based on the manufacturer’s instructions. The cells were further analyzed using flow cytometry (Cell Lab Quanta, Beckman Coulter, USA).

### Migration and invasion assays

The in vitro migration and invasion assays were performed using Transwell chambers. For the migration assay, 1 × 10^5^ TPC-1 or K1 cells with PRKCQ-AS1 overexpression were cultured in DMEM medium in the upper compartment of a Transwell chamber. For the invasion assay, 1 × 10^5^ TPC-1 or K1 cells with PRKCQ-AS1 silencing were resuspended in DMEM medium with 0.1% FBS in Matrigel-coated upper Transwell chambers. For both assays, the bottom chambers were filled with DMEM containing 10% FBS. The chambers were stained with 0.5% crystal violet. Migrated and invaded cells were counted under a microscope.

### In vivo animal experiments

All the animal experiments performed in this study were in accordance with the procedures approved by the institutional review board of Shanghai Tenth’s People Hospital. In this study, we used BALB/c mice at the age 4–6 weeks obtained from Shanghai Tenth’s People Hospital. These mice were housed under standard pathogen-free environments with 12 h dark and light cycles. Mice were subcutaneously injected with TPC-1 or K1 cells into the right flank of the mice. When the tumors were palpable, the mice were randomly assigned to the control group and the FMD group. In the control group, the average daily consumption of mice was 14.9 kJ. The FMD consisted of three components, designated as the day 1 diet, day 2–3 diet, and day 4–7 diet, fed in this order. The day 1 diet contained only 50% of the calories of the normal intake. The day 2–3 diet contained only 10% of the calories of the normal intake. For the day 4–7 diet, the mice were fed their normal intake; this progression was followed by another cycle of the FMD. The animals had free access to water. The mice were further monitored and the subcutaneous tumors were measured throughout the 5 weeks. At the end, the tumors were dissected and the tumors were imaged and weighed. The samples were either fixed for immunohistochemical analysis or frozen for western or RNA analysis.

### Immunohistochemistry

Immunohistochemical staining was performed on tumor tissue samples that are fixed in formalin and embedded in paraffin. After deparaffinization and rehydration, tissues were incubated with primary antibodies of Ki67 (Abcam) or IGF2BP1/2/3 (Abcam) at 4 °C overnight. Further, the tissues were treated with respective secondary antibodies. Finally, the sections were colored using diaminobenzidine before visualization under a light microscope (Leica DM IL, Germany).

### Fasting plasma glucose and insulin measurement

At the end of the xenograft model experiment, tail blood samples were collected after 10 h of food restriction and used for the measurement of blood glucose and insulin concentrations were measured by using a handheld glucometer (Yuwell, Jiangsu, China) and a murine enzyme-linked immunosorbent assay kit (Shanghai Enzyme-linked Biotechnology Co., Shanghai, China).

### Immunofluorescence staining

Cells were initially fixed using 4% paraformaldehyde for 15 minutes at room temperature. Further, after washing blocking of the samples were performed using 10% goat serum for 20 minutes at RT. Cells were then incubated with primary antibodies against IGF2BP1/2/3 overnight at 4 °C. Cells were thoroughly washed and incubated in respective secondary antibodies (Santa Cruz Biotechnology, USA) for 1 h at RT. Finally, the cells were stained with nuclear stain DAPI for 10 minutes before visualization using light microscope (Leica DM IL, Germany).

### JC-1 staining

Cells were initially seeded onto a 96-well plate and incubated overnight. Cells were further treated with serum-free medium containing Cis with/without 2 mM NAC. The cells were further washed and incubated with 5,5′,6,′-tetrachloro-1,1′,3,3′ tetra- ethylbenzimidazolyl carbocyanine iodide (JC-1, Beyotime, China) dye for half hour. The cells were finally washed and visualized using light microscope (Leica DM IL, Germany).

### RNA immunoprecipitation assay

TPC-1 or K1 cells were washed and lysed using RIP buffer at 4 °C for half hour. RIP assay was further performed based on manufacturer’s instructions (RIP lysis buffer, EMD Millipore, Billerica, MA, USA). The lysates were further treated with magnetic beads conjugated to the corresponding antibodies. The precipitated RNA was further isolated and quantified using qRT-PCR.

### Co-immunoprecipitation

To detect protein–protein interactions, cells were lysed in 500 μl co-IP buffer supplemented with a cocktail of proteinase inhibitors. The lysates were centrifuged at 12,000 × *g* for 30 min, and the supernatant was used for immunoprecipitation with beads, which were preincubated with the corresponding antibodies. After incubation at 4 °C overnight, beads were washed three times with co-IP buffer. SDS sample buffer was added to the beads and the immunoprecipitates were used for western blot analysis.

### Statistical analysis

All data shown in this study were analyzed using GraphPad Prism 8.0. Data are presented as mean ± standard error mean. Student T-test was used to assess statistical significance when comparing two groups. One-way analysis of variance was performed to assess significance when comparing multiple groups. *P* < 0.05 is considered as statistically significant.

### Supplementary information

Supplementary figure legends

Supplementary Table S1

Supplementary Figure S1

Supplementary Figure S2

Supplementary Figure S3

Supplementary Figure S4

Supplementary Figure S5

Supplementary Figure S6

Supplementary Figure S7

Supplementary Figure S8

Original western blots

## Data Availability

The datasets used and/or analyzed during the current study are available from the corresponding author on reasonable request.
