# Supplementary material for: Fasting regulates mitochondrial function through lncRNA PRKCQ-AS1-mediated IGF2BPs in papillary thyroid carcinoma
Source: Cell Death Dis. 2023 Dec 14;14(12):827. doi: 10.1038/s41419-023-06348-0 (PMC10719255; doi:10.1038/s41419-023-06348-0)
Supplement: Supplementary file 1 — Supplementary figure legends [file 41419_2023_6348_MOESM1_ESM.docx]

**Figure S1.** **Fasting impairs glycolysis in TPC-1 and K1 cells，which largely unaffected by TPC-1 BRAF^V600E^ and K1 RET/PTC1 rearrangements.** TPC-1 cells were treated with SU11248, a highly effective tyrosine inhibitor of the RET/PTC1 oncogenic kinase (1μM, 48h) before cultured in the fasting medium; while K1 cells were treated with Vemurafenib, a BRAF^V600E^ selective inhibitors (1μM, 48h) before cultured in the fasting medium. (**A-C**) ECAR, an indicator of glycolysis, and OCR, which reflects mitochondrial respiration, were measured in TPC-1 and K1 cells cultured in the fasting mimic medium for 48h. (**D-E**) Glucose uptake and lactate production via glycolysis in TPC-1 and K1 cells were detected. (**F**) The expression of rate-limiting glycolytic enzymes in glucose metabolism (PDK1, LDHA, HK2, GLUT1 and GPI) in TPC-1 and K1 cells were measured by western blotting. **P*<0.05, ***P*<0.01 and ****P*<0.001.

**Figure S2. Quantifying the basal respiration, ATP respiration and maximal respiration.** (**A**) Scheme demonstrating the breakdown of OCR. Maximal respiration is the OCR after the injection of FCCP minus the OCR after injection of oligomycin. ATP respiration (based on respiration) is the difference between the basal respiration and the respiration after the injection of oligomycin. (**B**) Quantifying the basal respiration, ATP respiration and maximal respiration in Figure 1E. (**C**) Quantifying the basal respiration, ATP respiration and maximal respiration in Figure 5G. **(D**) Quantifying the basal respiration, ATP respiration and maximal respiration in Figure 8D. **P*<0.05, ***P*<0.01 and ****P*<0.001.

**Figure S3. Body weight, fasting blood glucose and insulin concentrations were detected *in vivo* animal experiments of fasting mimic diet model.** TPC-1 and K1 cells were injected into BALB/c mice. When the tumors were palpable, the mice were randomly assigned to the control group or the fasting mimic diet (FMD) group. (**A**) The body weights of mice were measured every week and a weight profile during the FMD cycle was established. Mice in the FMD group lost 10% of their body weight during each FMD cycle but regained most of their body weight after refeeding, n = 6. (**B-C**) Blood samples were collected on the last day of experiments, and fasting blood glucose and insulin levels were detected, n = 6. **P*<0.05 and ***P*<0.01.

**Figure S4. (A)** The expression of PRKCQ-AS1 was decreased significantly in the papillary thyroid cancer compared with that in the normal group in the GSE 3678 data set (n=7). The relative expression of PRKCQ-AS1 were quantified presented below. **(B)** The expression of PRKCQ-AS1 was decreased significantly in the papillary thyroid cancer compared with that in the normal group in the GSE 3467 data set (n=9). The relative expression of PRKCQ-AS1 were quantified presented below. ***P*<0.01 and ****P*<0.001.

**Figure S5. IGF2BP1 has almost no difference in PTC tissues and cells, compared to normal tissues. (A)** The expression of IGF2BP1 in THCA from TCGA database were shown. **(B)** MRNA expression of IGF2BP1 was determined by qRT-PCR in PTC tissues (n=107). (**C**) MRNA expression of IGF2BP1 was determined by RT-PCR in PTC cells (n=3). **P*<0.05.

**Figure S6**. **IGF2BP2/3 regulates PTC cell cycle in TPC-1 and K1 cells.** (**A**) qRT-PCR analysis of IGF2BP2/3 expression levels in TPC-1 transfected with vector/IGF2BP2/3 and in K1 cells transfected with sh-NC/sh-IGF2BP2/3 (**B**) The cell cycle distribution was analyzed by flow cytometry analysis in TPC-1 and K1 cells. TPC-1 cells were tranfected with Vector/IGF2BP2/3 and K1 cells were transfected with sh-NC or sh-IGF2BP2/3 overexpression. ***P*<0.01 and ****P*<0.001.

**Figure. S7. PRMT7 knockdown inhibits PRMT7 expression; PRMT7 overexpression has the opposite effect in TPC-1 and K1 cells. (A)** The mRNA and protein expression levels were determined by qRT-PCR and western blot in TPC-1 and K1 cells, which were transfected with pcDNA3.1/PRMT7 or shScramble/shPRMT7. **P*<0.05, ***P*<0.01 and ****P*<0.001.

**Figure S8.** **Fasting regulates lncRNA PRKCQ-AS1/IGF2BPs/PRMT7 signaling pathway** **in** **papillary thyroid carcinoma. (A-B)** TPC-1 cells transfected with vector, PRKCQ-AS1, and PRKCQ-AS1+IGF2BP2 were cultured in the control medium and fasting medium for 48h; K1 cells transfected with sh-NC, sh-PRKCQ-AS1 and sh-PRKCQ-AS1+sh-IGF2BP2 were cultured in the control medium and fasting medium for 48h. Protein and mRNA expression of IGF2BP2 and PRMT7 were determined by western blot and qTR-PCR analysis. (**C-D**) TPC-1 cells transfected with vector, PRKCQ-AS1, and PRKCQ-AS1+IGF2BP3 were cultured in the control medium and fasting medium for 48h; K1 cells transfected with sh-NC, sh-PRKCQ-AS1 and sh-PRKCQ-AS1+sh-IGF2BP2 were cultured in the control medium and fasting medium for 48h. Protein and mRNA expression of IGF2BP3 and PRMT7 were analyzed by western blot and qTR-PCR. **P*<0.05, ***P*<0.01, ****P*<0.001 and ^&^*^&^P*<0.01, ^&&&^*P*<0.001.
