# Supplementary material for: Fasting regulates mitochondrial function through lncRNA PRKCQ-AS1-mediated IGF2BPs in papillary thyroid carcinoma
Source: Cell Death Dis. 2023 Dec 14;14(12):827. doi: 10.1038/s41419-023-06348-0 (PMC10719255; doi:10.1038/s41419-023-06348-0)
Supplement: Supplementary file 2 — Supplementary Table S1 [file 41419_2023_6348_MOESM2_ESM.docx]

| **Table S1. Information on the RT-PCR primer sequence** | | |
| --- | --- | --- |
| Name | Primer direction | Sequence |
| PRKCQ-AS1 | Forward | 5’-GTCAGGAAAAGGGGCGAGAG-3’ |
|  | Reverse | 5’-CAAATGCCTGGGGTAGGTCA-3 |
| IGF2BP1 | Forward | 5’- CTCTCGGAGGGGTTTCGGA -3’ |
|  | Reverse | 5’- CTCTCGTTGAGGTTGCCGAT -3’ |
| IGF2BP2 | Forward | 5’- CGGGGAAGAGACGGATGATG -3’ |
|  | Reverse | 5’- GTGTCTGTGTTGACTTGTTCCA -3’ |
| IGF2BP3 | Forward | 5’- GGATGCGTTTGGGTTGTAGC -3’ |
|  | Reverse | 5’- ACTATCCAGCACCTCCCACT -3’ |
| PRMT7 | Forward | 5’- AAAGTGGTAGCAGCGGAGG -3’ |
|  | Reverse | 5’- AGCTTGTTCCACGACCACAT -3’ |
| GAPDH | Forward | 5’- TTGGTATCGTGGAAGGACTCA -3’ |
|  | Reverse | 5’- TGTCATCATATTTGGCAGGTT -3’ |
| PGK1 | Forward | 5’- TTGACCGAATCACCGACCTC -3’ |
|  | Reverse | 5’-CATAACGACCCGCTTCCCTT -3’ |
| LDHA | Forward | 5’-AGGCTACACATCCTGGGCTA -3’ |
|  | Reverse | 5’-GTACAAGAAAGTTGGGTAAAATTGC-3’ |
| HK2 | Forward | 5’- TGTGAATCGGAGAGGTCCCA -3’ |
|  | Reverse | 5’- CCCAAAGCACACGGAAGTTG-3’ |
| GLUT1 | Forward | 5’- TTCACTGTCGTGTCGCTGTT-3’ |
|  | Reverse | 5’-TACTGGAAGCACATGCCCAC -3’ |
| GPI | Forward | 5’- ACCACCAGCAGACACACATC-3’ |
|  | Reverse | 5’- AGACAGGGCAACAAAGTGCT-3’ |
| MFN1 | Forward | 5’- -AACCACCAAGGAGTGTGGAA3’ |
|  | Reverse | 5’-TCCCTCCCATGAAAAGGAAAC -3’ |
| MFN2 | Forward | 5’-GTGAAGTCAGGACTGGTGGA -3’ |
|  | Reverse | 5’-TCGAGAGAAGAGCAGGGACA -3’ |
| OPA1 | Forward | 5’-CTGTGGCCTGTGAGGTCTG -3’ |
|  | Reverse | 5’-AATAGGGCCACATGGTGAGG -3’ |
| DRP1 | Forward | 5’-AGAAAATGGGGTGGAAGCAGA -3’ |
|  | Reverse | 5’-AGGACGAGGACCAGTAGCAT -3’ |
| FIS1 | Forward | 5’- CTGCTCCCCTGAGATTCGTC-3’ |
|  | Reverse | 5’-AGCCACAGCCCCGTTTTATT -3’ |
| MFF | Forward | 5’-CGTGCTCTCAGCCAACCA -3’ |
|  | Reverse | 5’- TGCCAACTGCTCGGATTTCT-3’ |
