# Supplementary figures and images for: Fasting regulates mitochondrial function through lncRNA PRKCQ-AS1-mediated IGF2BPs in papillary thyroid carcinoma
Source: Cell Death Dis. 2023 Dec 14;14(12):827. doi: 10.1038/s41419-023-06348-0 (PMC10719255; doi:10.1038/s41419-023-06348-0)

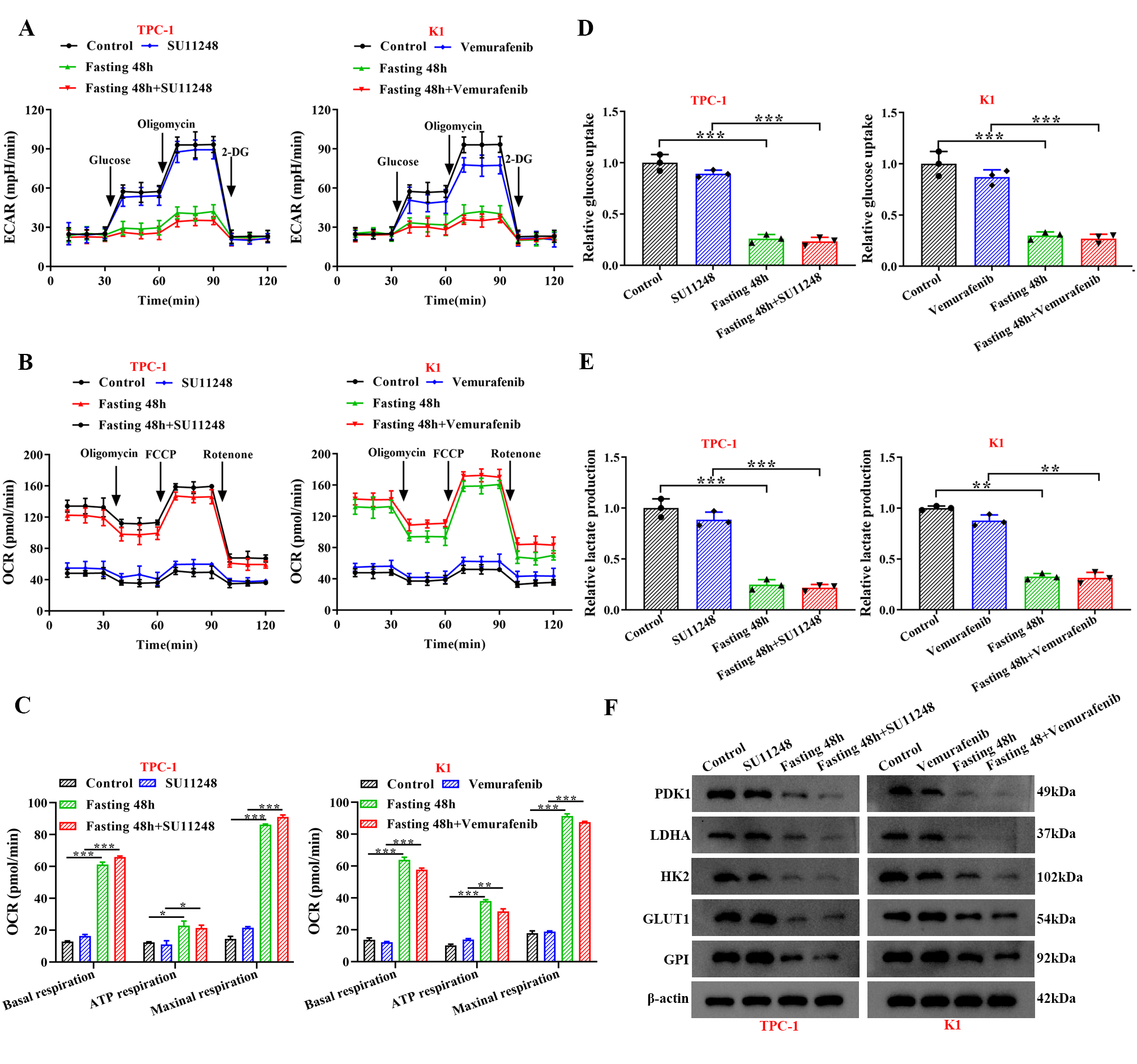

Supplement: Supplementary file 3 — Supplementary Figure S1 [file 41419_2023_6348_MOESM3_ESM.tif]

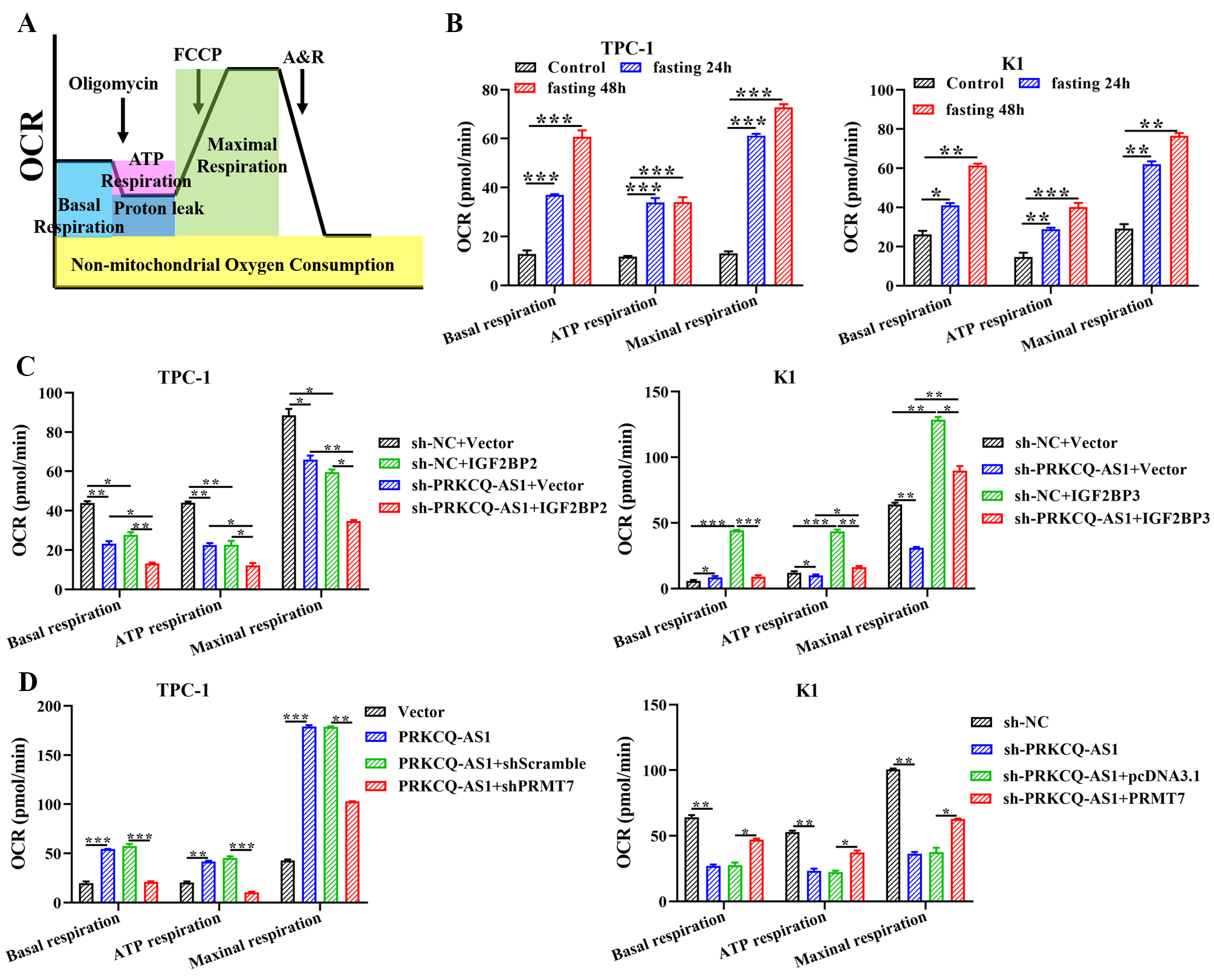

Supplement: Supplementary file 4 — Supplementary Figure S2 [file 41419_2023_6348_MOESM4_ESM.tif]

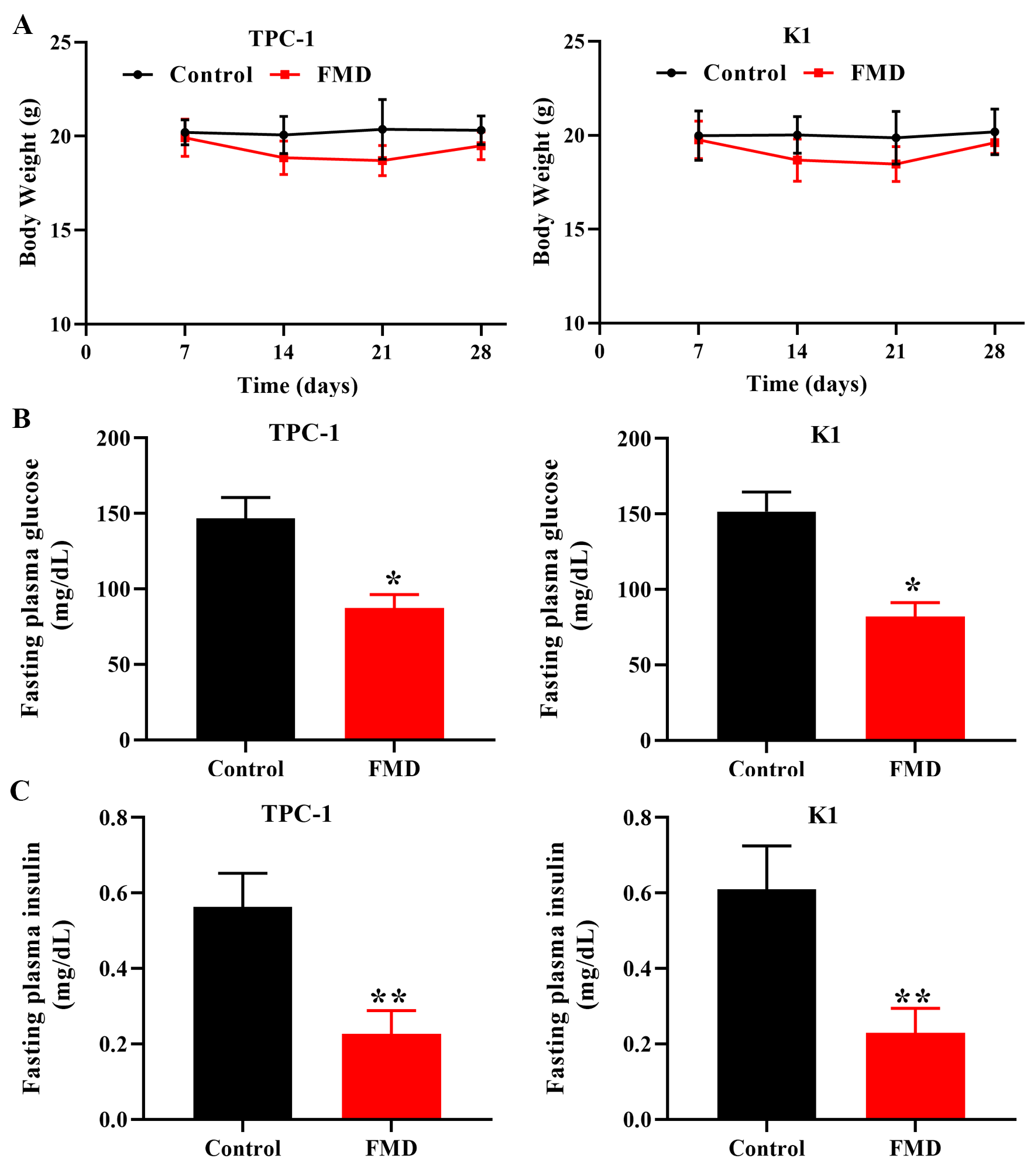

Supplement: Supplementary file 5 — Supplementary Figure S3 [file 41419_2023_6348_MOESM5_ESM.tif]

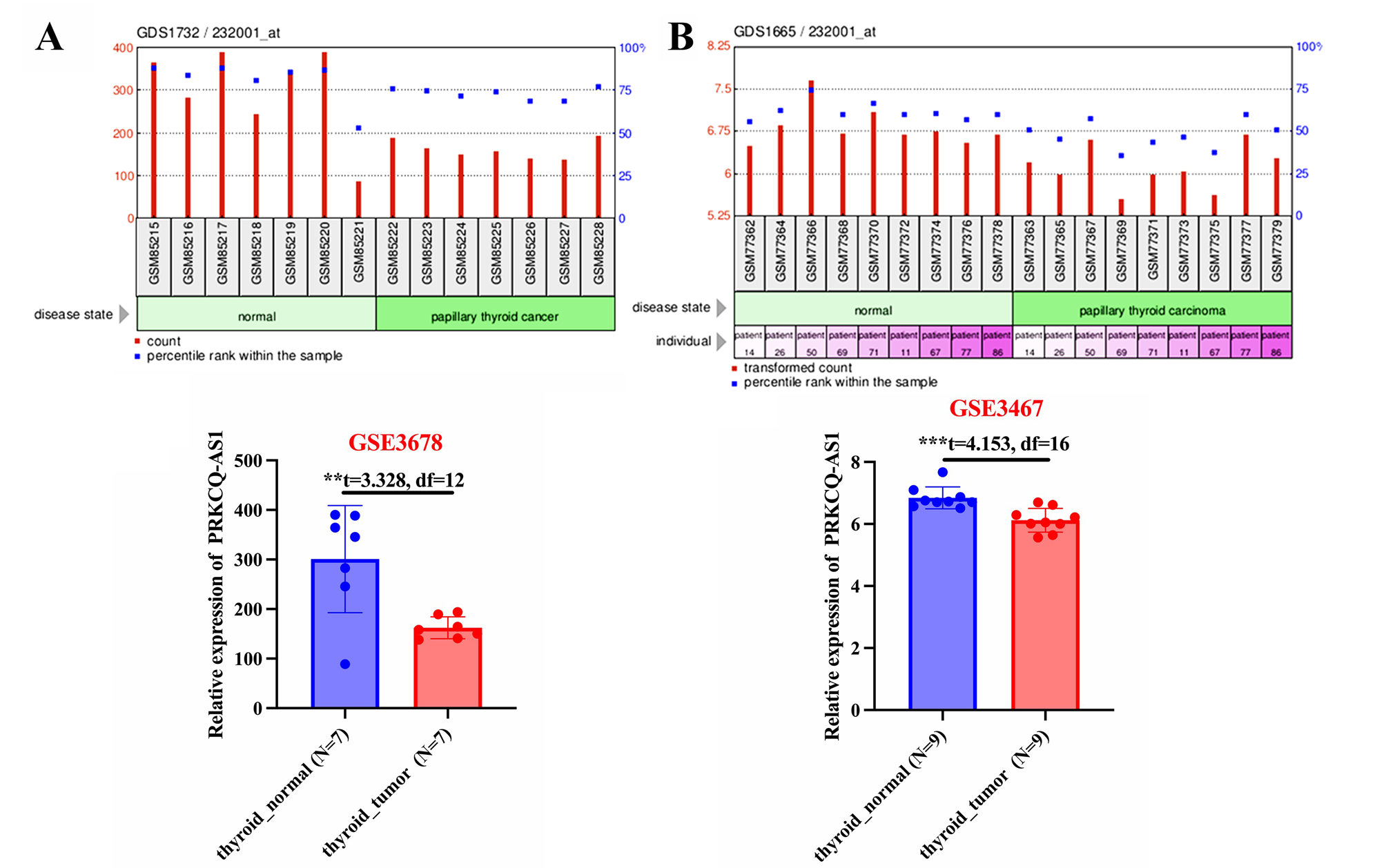

Supplement: Supplementary file 6 — Supplementary Figure S4 [file 41419_2023_6348_MOESM6_ESM.tif]

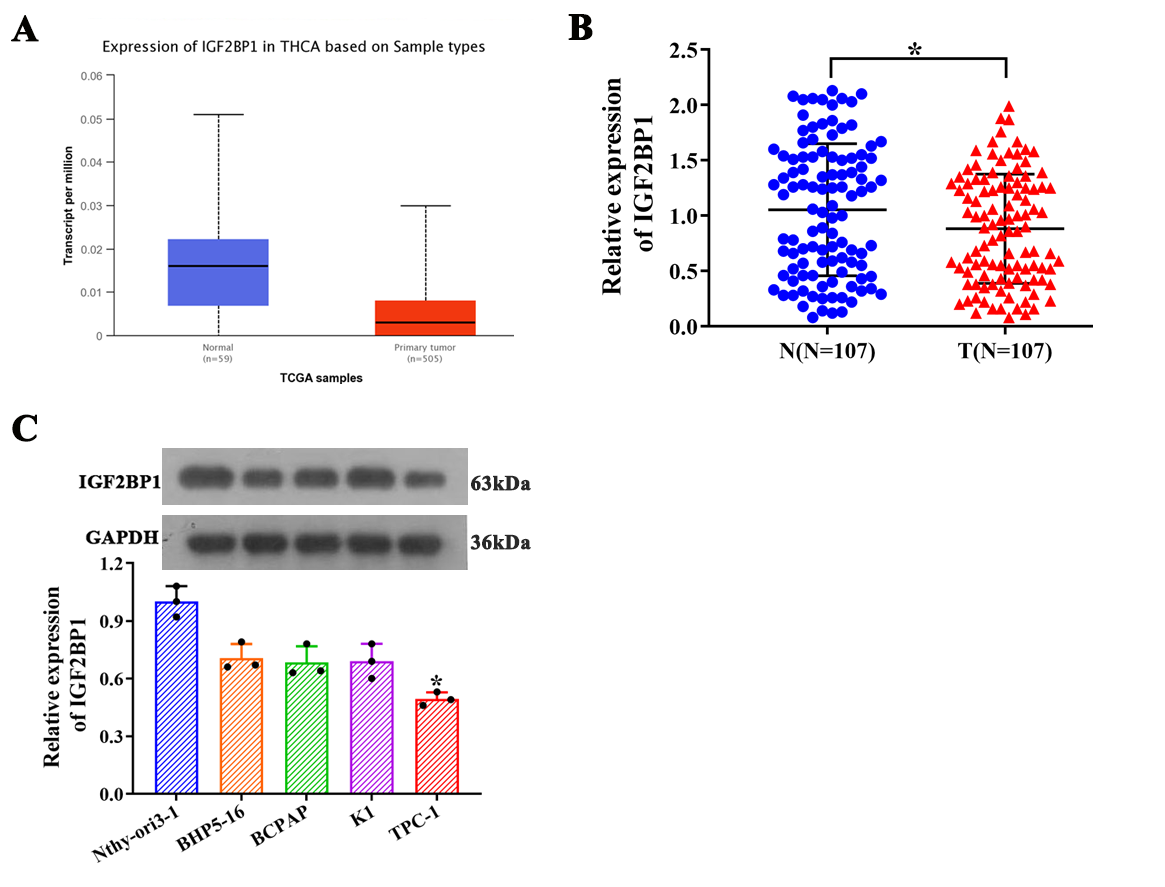

Supplement: Supplementary file 7 — Supplementary Figure S5 [file 41419_2023_6348_MOESM7_ESM.tif]

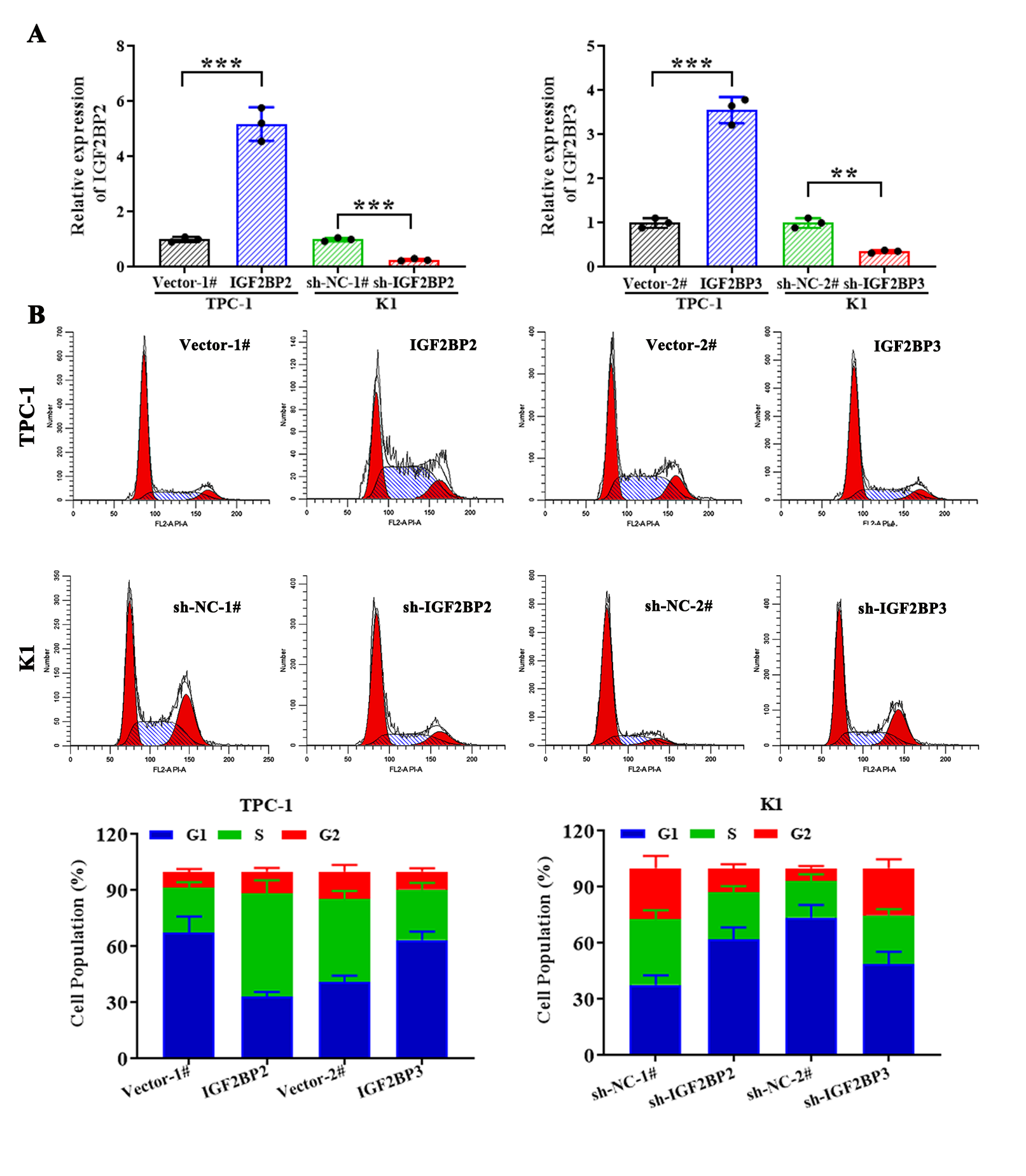

Supplement: Supplementary file 8 — Supplementary Figure S6 [file 41419_2023_6348_MOESM8_ESM.tif]

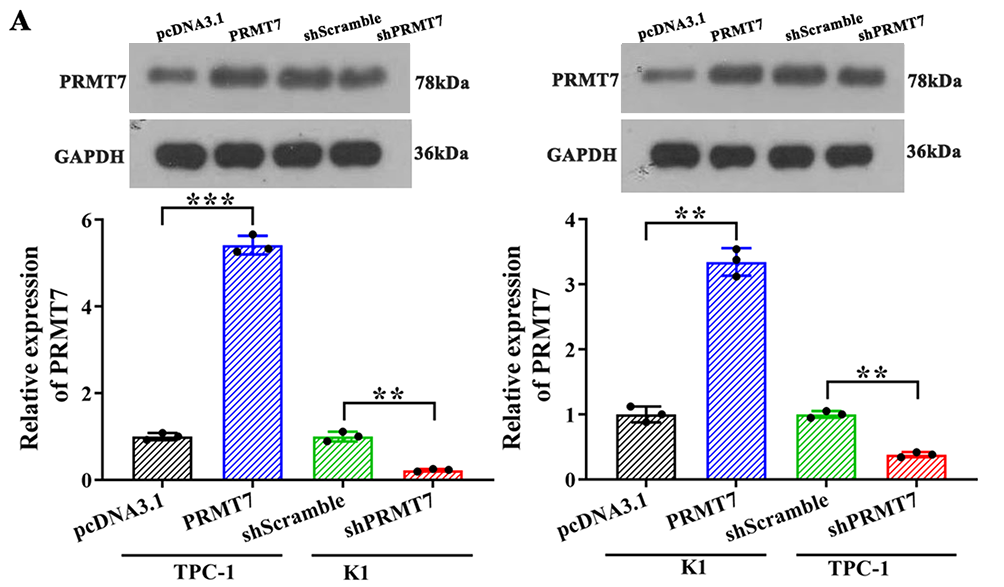

Supplement: Supplementary file 9 — Supplementary Figure S7 [file 41419_2023_6348_MOESM9_ESM.tif]

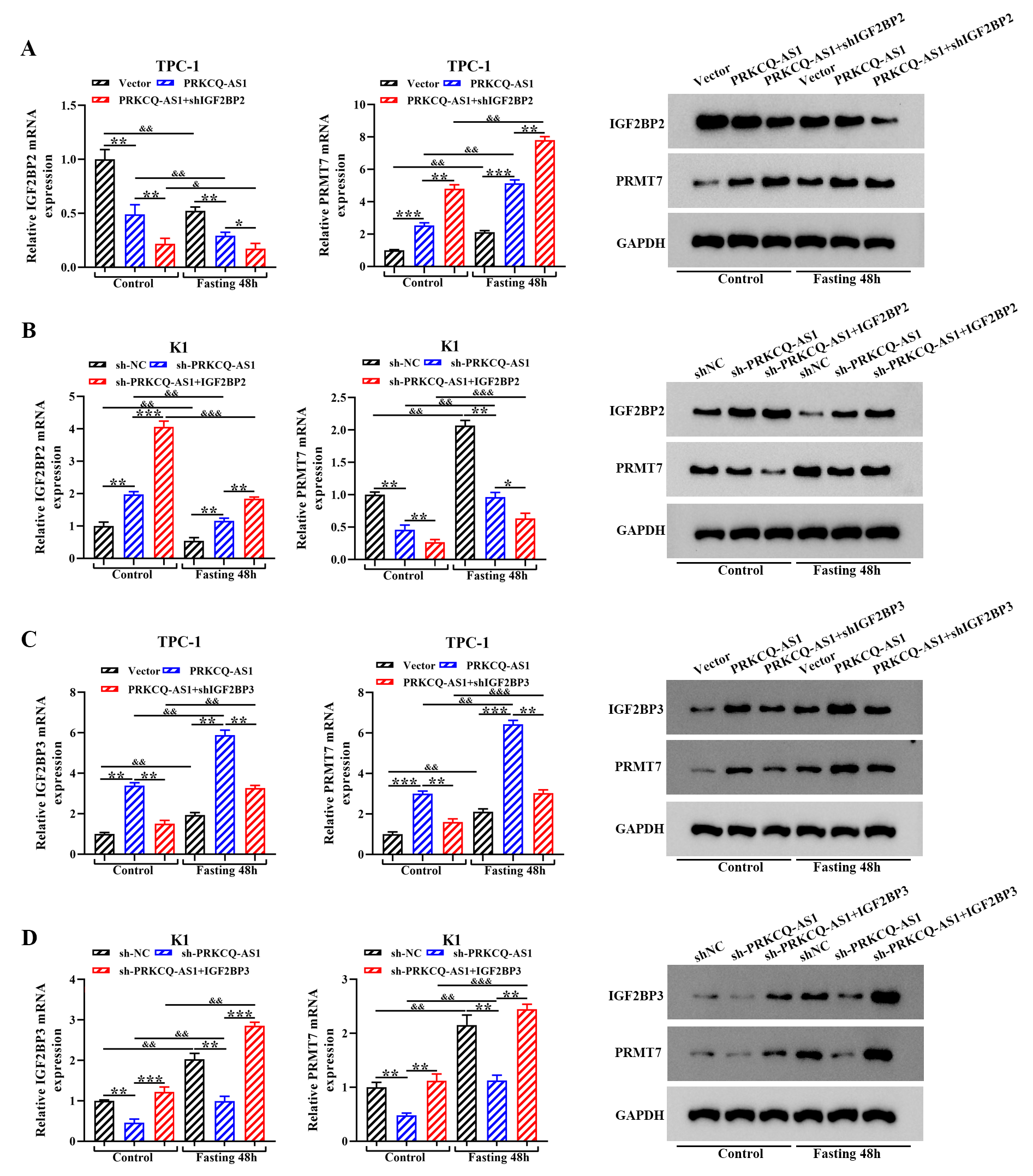

Supplement: Supplementary file 10 — Supplementary Figure S8 [file 41419_2023_6348_MOESM10_ESM.tif]
