# Supplementary material for: Fasting regulates mitochondrial function through lncRNA PRKCQ-AS1-mediated IGF2BPs in papillary thyroid carcinoma
Source: Cell Death Dis. 2023 Dec 14;14(12):827. doi: 10.1038/s41419-023-06348-0 (PMC10719255; doi:10.1038/s41419-023-06348-0)

Figure 1F

TPC-1 | **K1**

PDK1

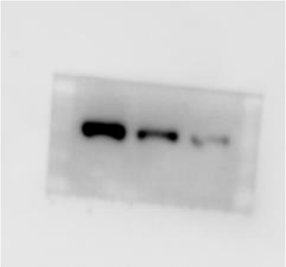

PDK1

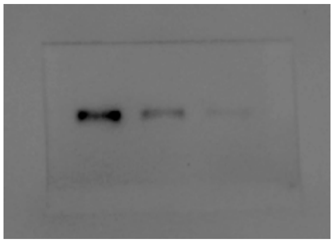

LDHA

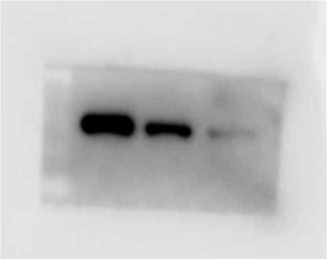

LDHA

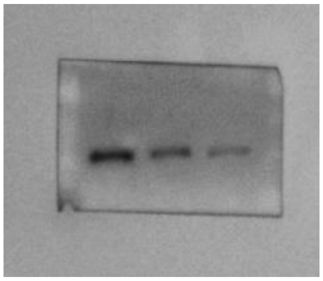

HK2

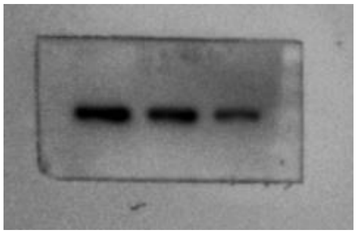

HK2

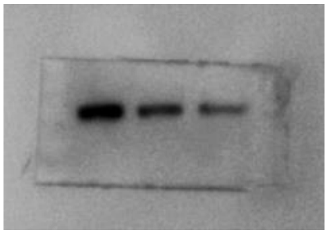

GLUT1

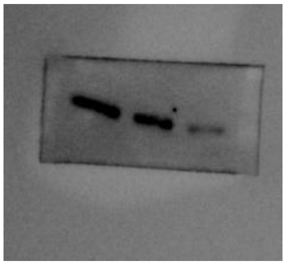

GLUT1

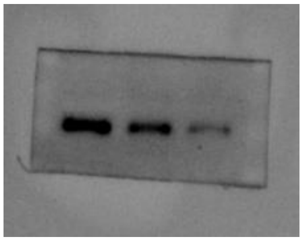

GPI

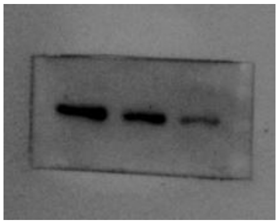

GPI

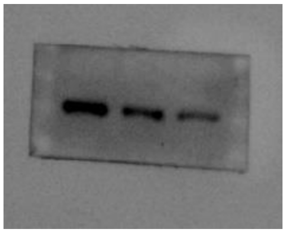

β-actin β-actin

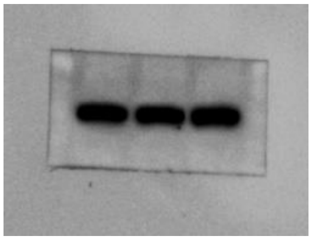

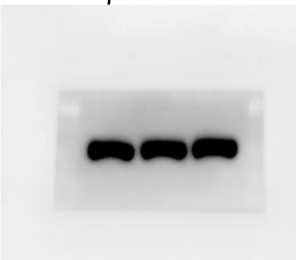

Figure 1J

TPC-1 K1

MFN1 MFN1

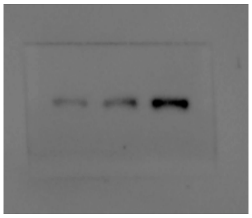

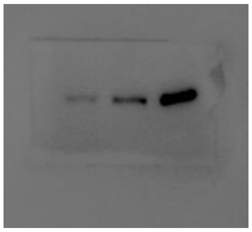

MFN2 MFN2

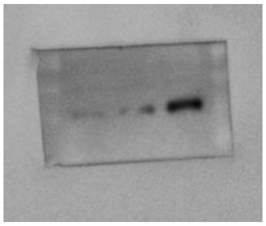

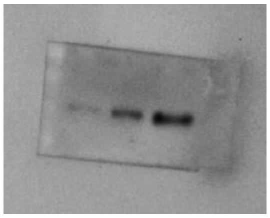

OPA1 OPA1

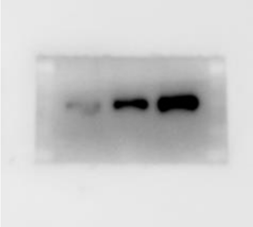

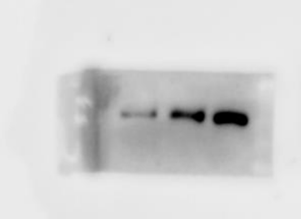

DRP1 DRP1

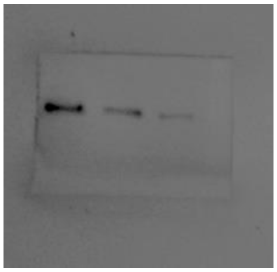

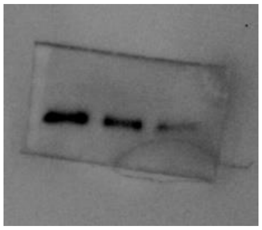

FIS1

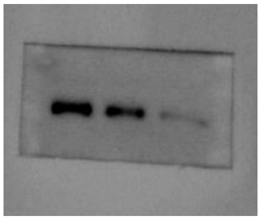

FIS1

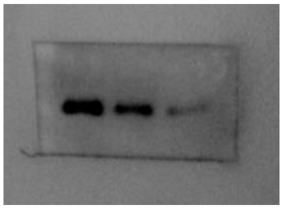

MFF

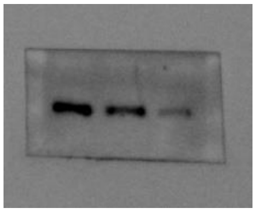

MFF

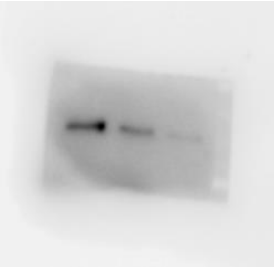

β-actin

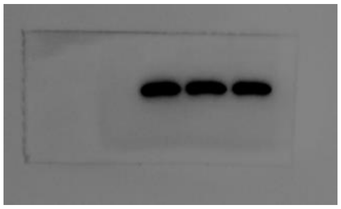

β-actin

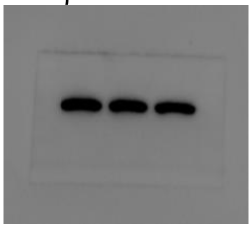

Figure 4E

IGF2BP1

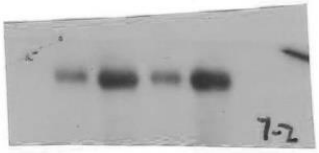

IGF2BP2

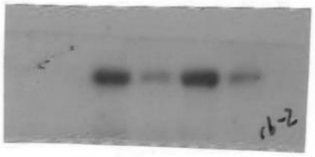

IGF2BP3

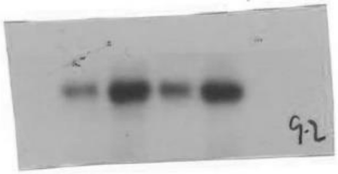

GAPDH

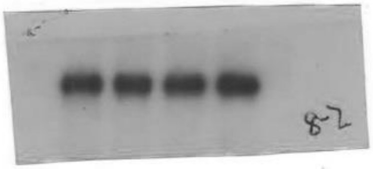

Figure 4F

IGF2BP1

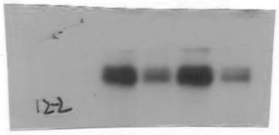

IGF2BP2

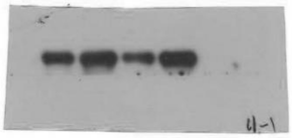

IGF2BP3

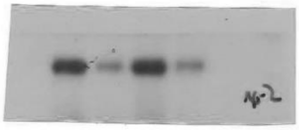

GAPDH

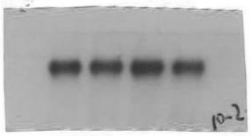

Figure 4J

IGF2BP2

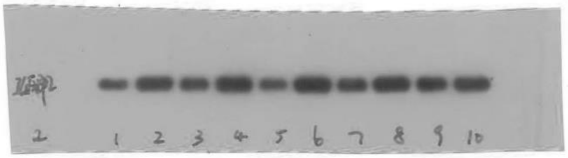

IGF2BP3

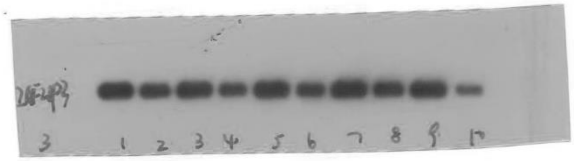

GAPDH

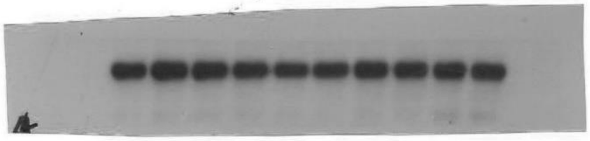

Figure 5A

IGF2BP2

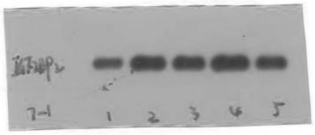

GAPDH

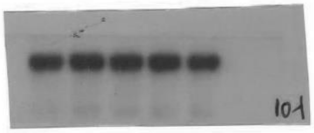

IGF2BP3

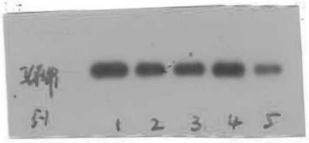

GAPDH

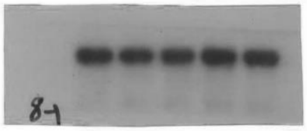

Figure 5H

TPC-1
PDK1

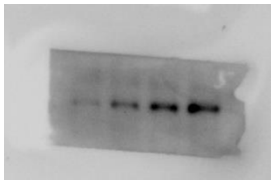

K1
PDK1

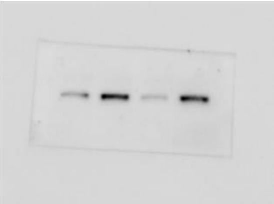

LDHA

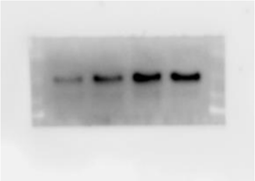

LDHA

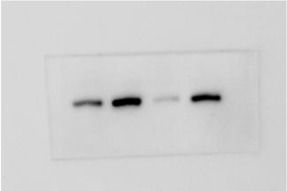

HK2

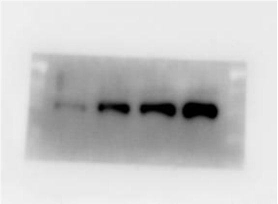

HK2

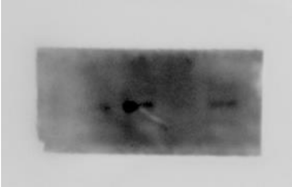

GLUT1

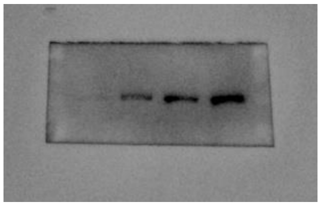

GLUT1

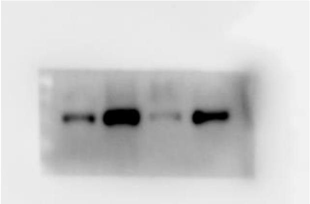

GPI GPI

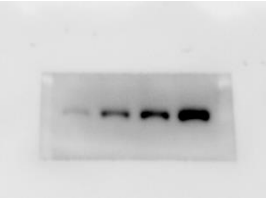

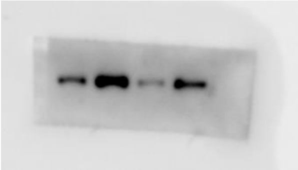

β-actin β-actin

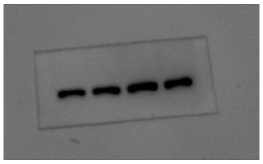

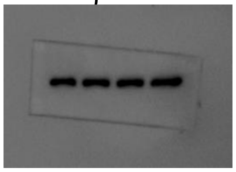

Figure 5L

TPC-1 **K1**

MFN1 MFN1

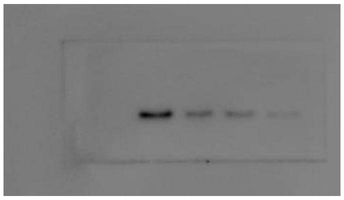

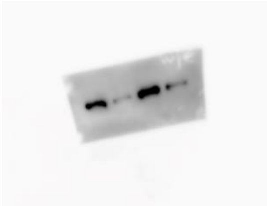

MFN2 MFN2

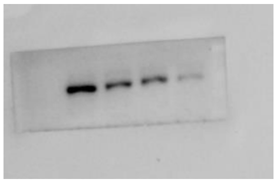

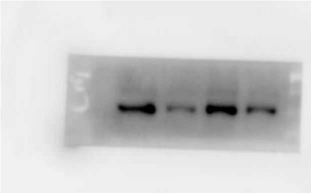

OPA1 OPA1

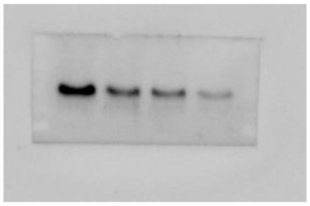

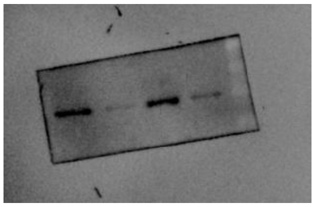

DRP1 DRP1

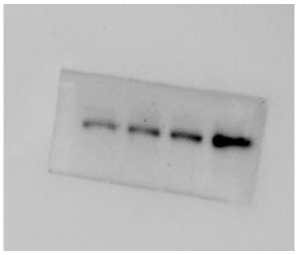

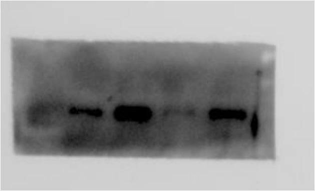

FIS1

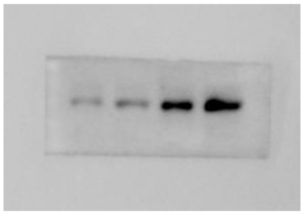

FIS1

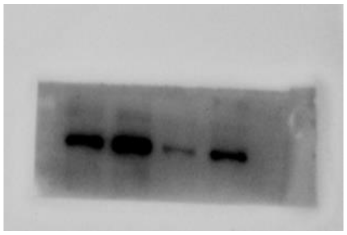

MFF

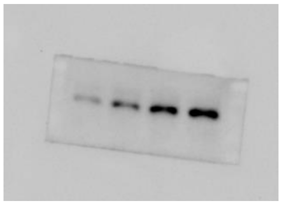

MFF

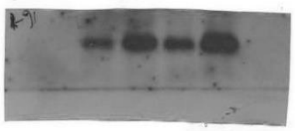

β-actin

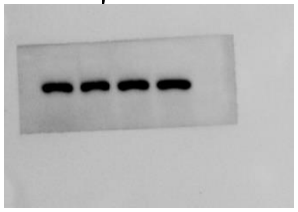

β-actin

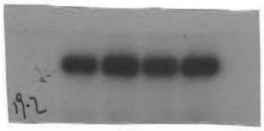

Figure 6E

TPC-1

PRMT7 GAPDH

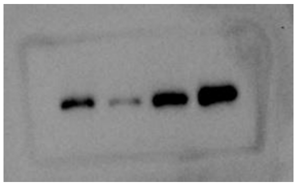

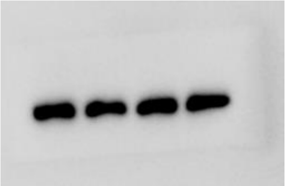

K1

PRMT7 GAPDH

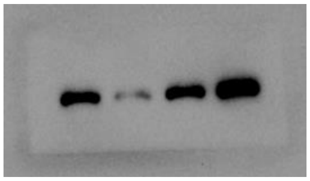

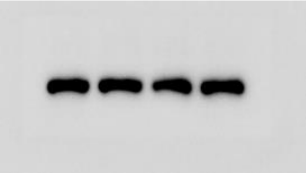

Figure 6F

TPC-1

PRMT7 GAPDH

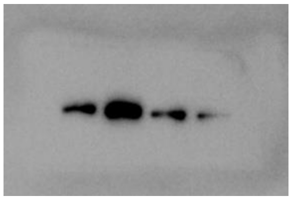

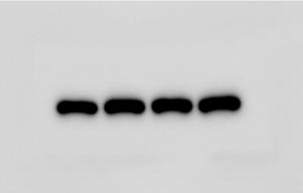

K1

PRMT7 GAPDH

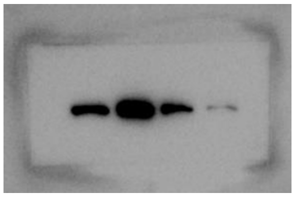

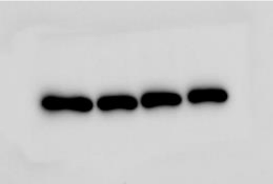

Figure 6G

TPC-1

PRMT7 GAPDH

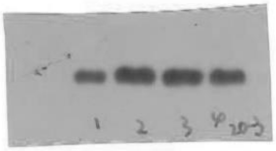

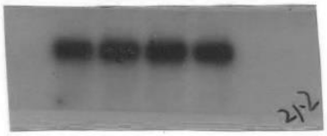

K1

PRMT7

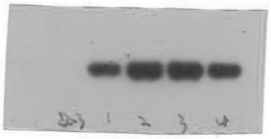

GAPDH

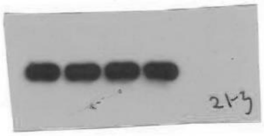

Figure 6I

TPC-1

PRMT7

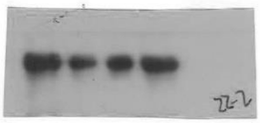

PRMT7

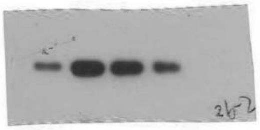

GAPDH

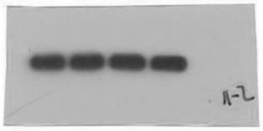

GAPDH

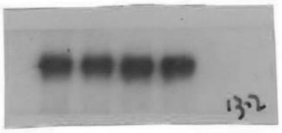

K1

PRMT7

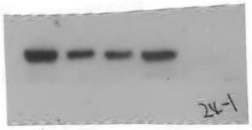

PRMT7

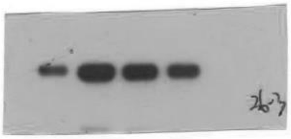

GAPDH

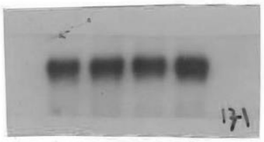

GAPDH

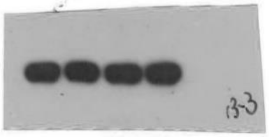

Figure 6J

TPC-1

PRMT7

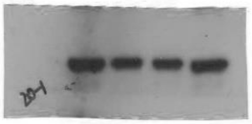

PRMT7

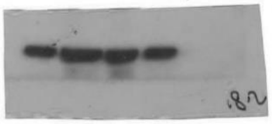

GAPDH

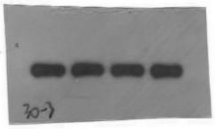

GAPDH

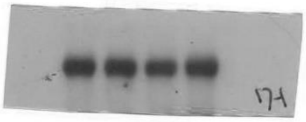

K1

PRMT7

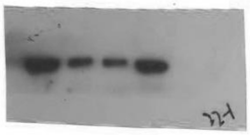

PRMT7

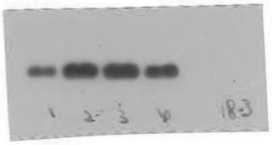

GAPDH

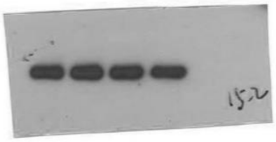

GAPDH

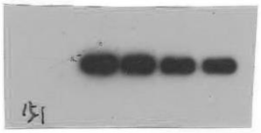

Figure 7C

PRMT7

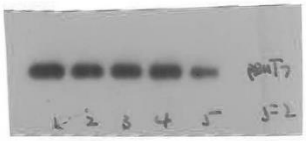

GAPDH

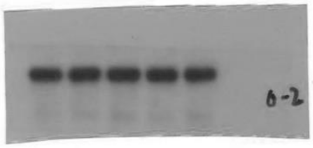

Figure 8E

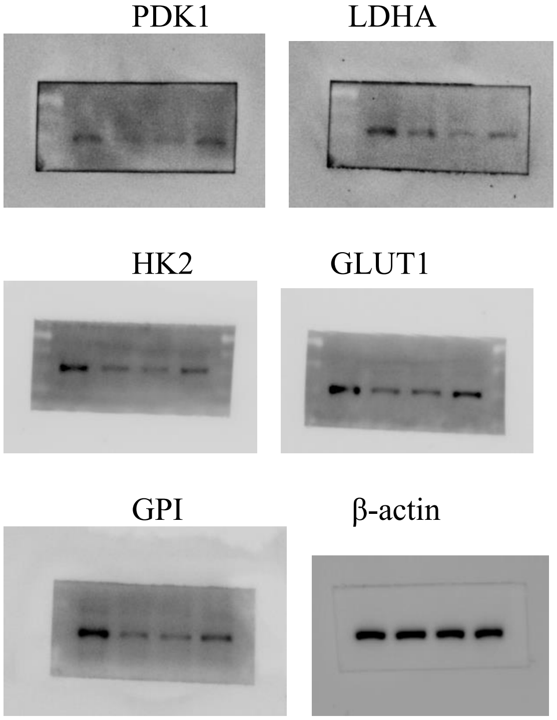

Figure 8F

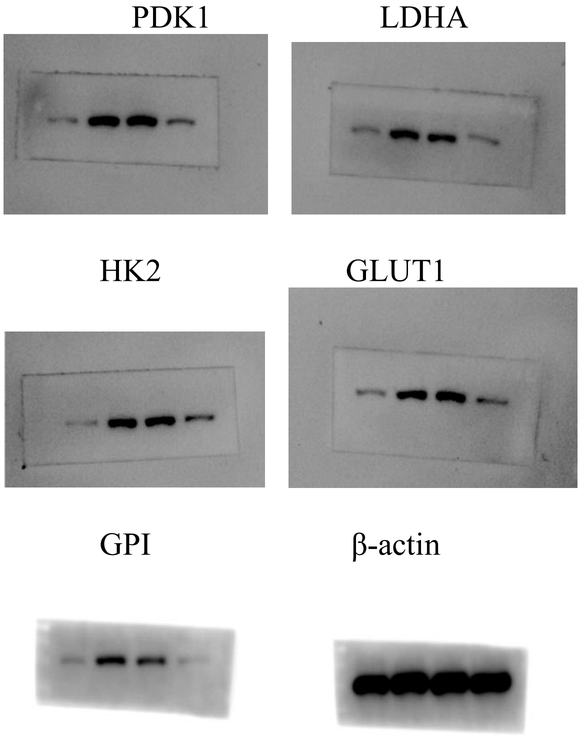

Figure 8L

Left | **right**

MFN1 | MFN1

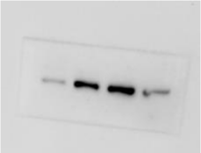

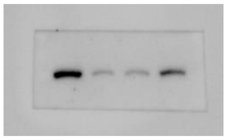

MFN2 | MFN2

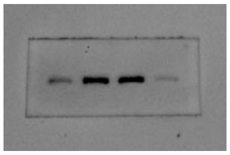

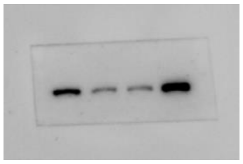

OPA1 | OPA1

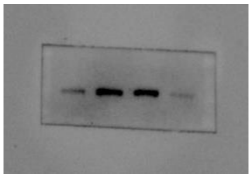

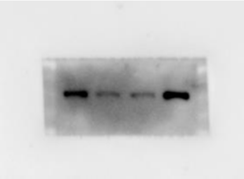

DRP1 | DRP1

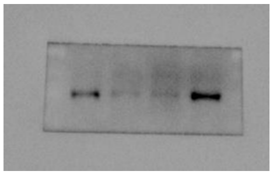

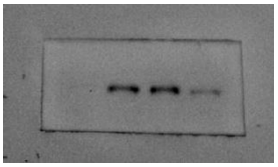

FIS1 | FIS1

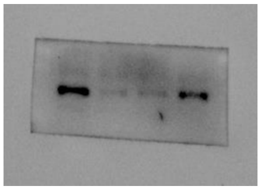

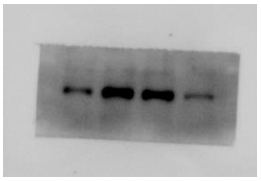

MFF | MFF

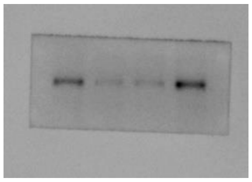

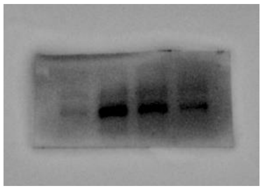

β-actin | β-actin

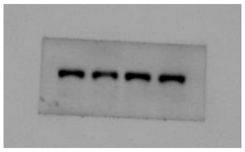

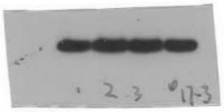

Figure S1F

TPC-1 **K1**

PDK1

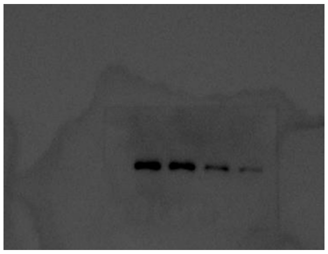

PDK1

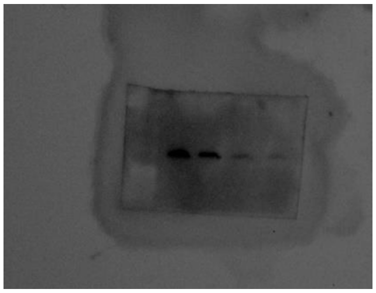

LDHA

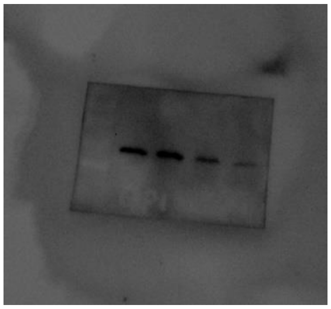

LDHA

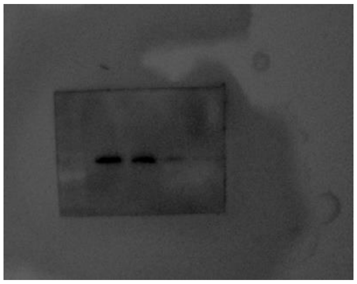

HK2

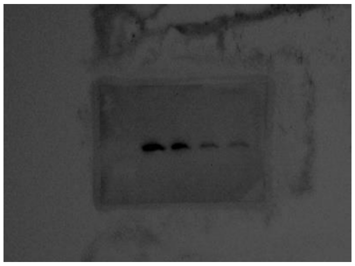

HK2

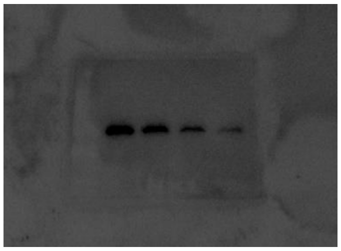

GLUT1

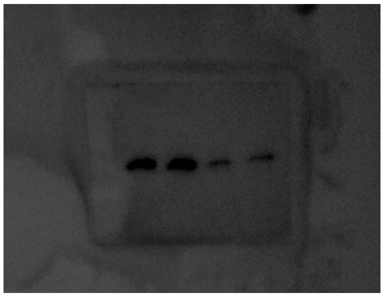

GLUT1

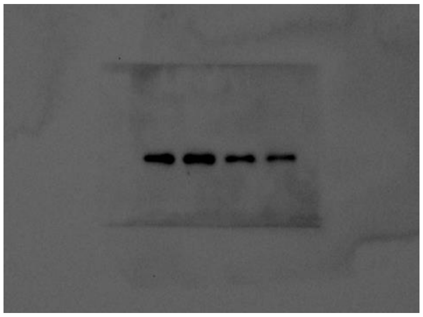

GPI

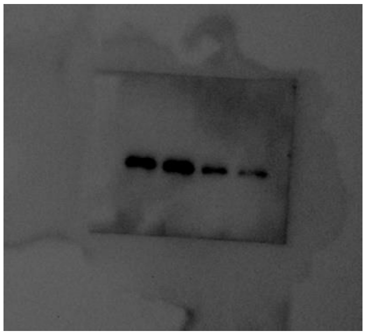

GPI

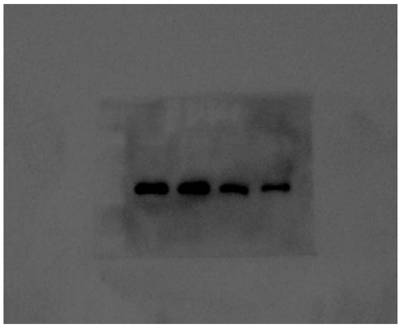

β-actin

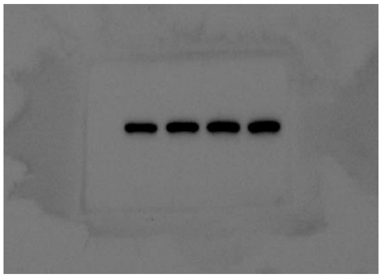

β-actin

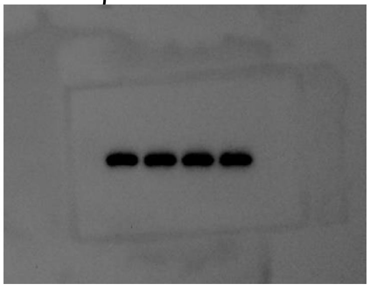

Figure S5C

IGF2BP1

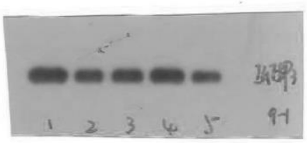

GAPDH

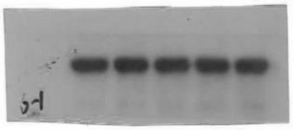

Figure S7A

Left

PRMT7

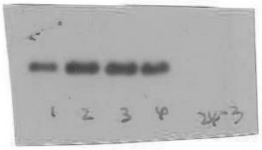

GAPDH

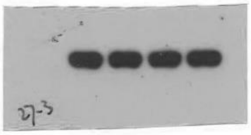

Right

PRMT7

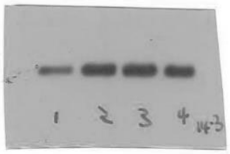

GAPDH

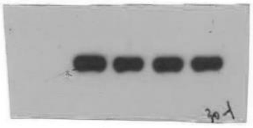

Figure S8A

TPC-1

IGF2BP2

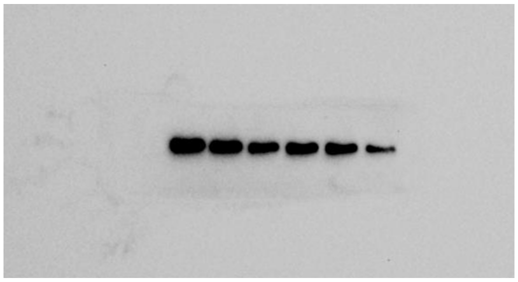

PRMT7

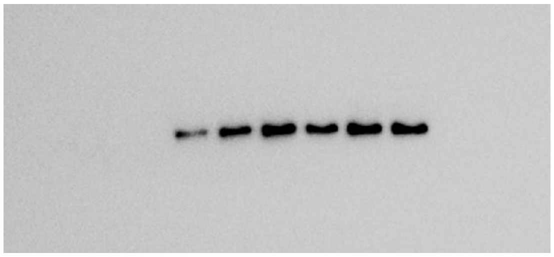

GAPDH

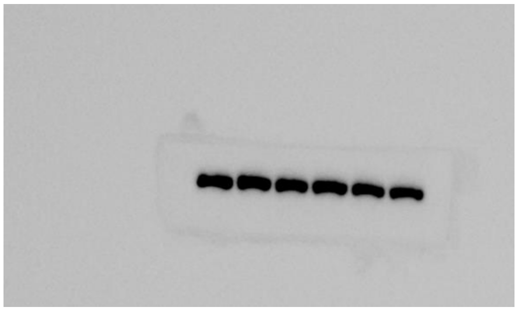

Figure S8B

K1

IGF2BP2

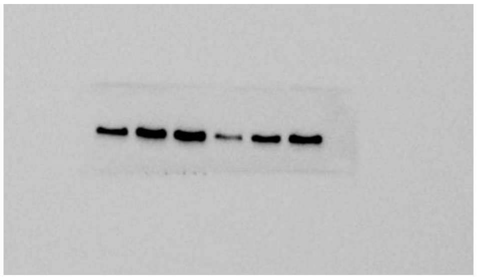

PRMT7

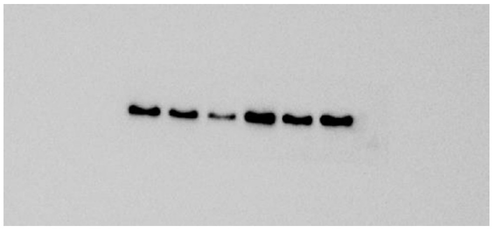

GAPDH

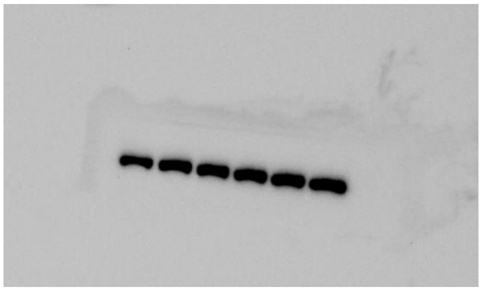

Figure S8C

TPC-1

IGF2BP3

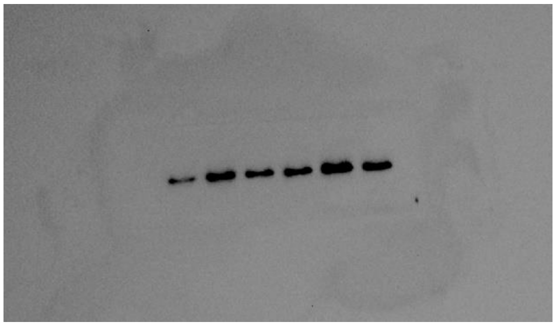

PRMT7

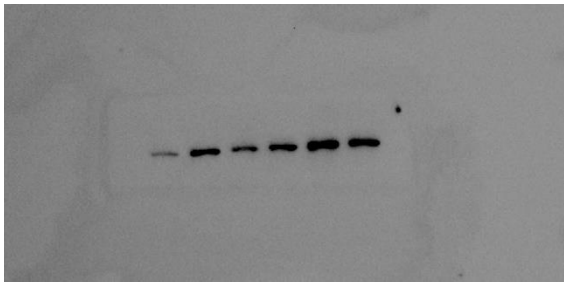

GAPDH

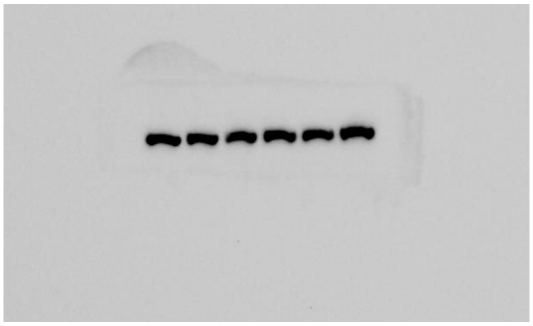

Figure S8D

K1

IGF2BP3

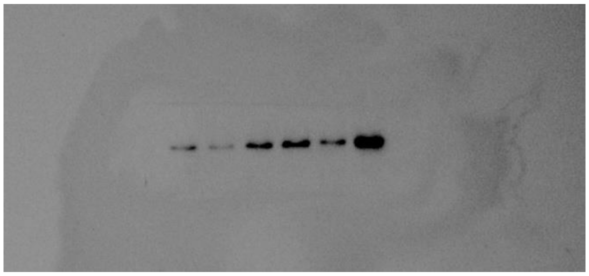

PRMT7

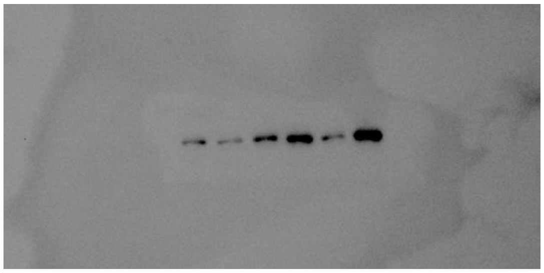

GAPDH

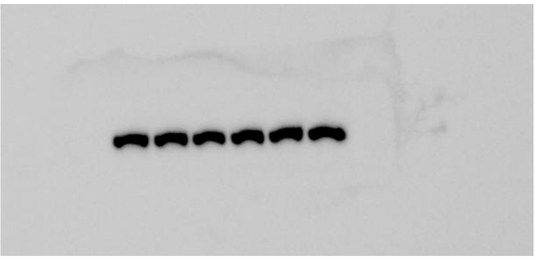

Supplement: Supplementary file 11 — Original western blots [file 41419_2023_6348_MOESM11_ESM.pdf]
